# Immobilized Lignin Peroxidase-Like Metalloporphyrins as Reusable Catalysts in Oxidative Bleaching of Industrial Dyes

**DOI:** 10.3390/molecules21070964

**Published:** 2016-07-22

**Authors:** Paolo Zucca, Cláudia M. B. Neves, Mário M. Q. Simões, Maria da Graça P. M. S. Neves, Gianmarco Cocco, Enrico Sanjust

**Affiliations:** 1 Dipartimento di Scienze Biomediche, Università di Cagliari, Complesso Universitario, SP1 Km 0.700, Monserrato (CA) 09042, Italy; pzucca@unica.it or paolo.zucca@consorziouno.it (P.Z.); gmarco.cocco@unica.it (G.C.); 2 Consorzio UNO Oristano, via Carmine snc, Oristano 09170, Italy; 3 Department of Chemistry and QOPNA, University of Aveiro, Aveiro 3810-193, Portugal; claudianeves@ua.pt (C.M.B.N.); msimoes@ua.pt (M.M.Q.S.); gneves@ua.pt (M.G.P.M.S.N.)

**Keywords:** metalloporphyrins, wastewaters, textile dyes, biomimetic, lignin peroxidase, immobilization

## Abstract

Synthetic and bioinspired metalloporphyrins are a class of redox-active catalysts able to emulate several enzymes such as cytochromes P450, ligninolytic peroxidases, and peroxygenases. Their ability to perform oxidation and degradation of recalcitrant compounds, including aliphatic hydrocarbons, phenolic and non-phenolic aromatic compounds, sulfides, and nitroso-compounds, has been deeply investigated. Such a broad substrate specificity has suggested their use also in the bleaching of textile plant wastewaters. In fact, industrial dyes belong to very different chemical classes, being their effective and inexpensive oxidation an important challenge from both economic and environmental perspective. Accordingly, we review here the most widespread synthetic metalloporphyrins, and the most promising formulations for large-scale applications. In particular, we focus on the most convenient approaches for immobilization to conceive economical affordable processes. Then, the molecular routes of catalysis and the reported substrate specificity on the treatment of the most diffused textile dyes are encompassed, including the use of redox mediators and the comparison with the most common biological and enzymatic alternative, in order to depict an updated picture of a very promising field for large-scale applications.

## 1. Introduction

Textile industry is one of the largest water-consuming industrial sectors [1], releasing huge amounts of colored (absorbance up to 200), polluting (Chemical Oxygen Demand–COD, up to 60,000 ppm), and highly recalcitrant wastes in the environment.

It has been estimated that every year more than 7 × 10^5^ tons of dyes are produced, and about 15% of the total amount used is released in the environment due to the low yields of textile processes [2,3]. In addition to the textile industry, dyes are also used by other sectors, such as food, cosmetic, paper, photographic, and plastic industries.

The US Environmental Protection Agency (US-EPA) suggests dividing textile wastes into four principal categories: (i) dispersible; (ii) hard-to-treat; (iii) high-volume; (iv) hazardous and toxic wastes [4]. Their environmental impact is firstly evident from an aesthetical perspective (dyes absorb light in the visible range, being detectable even at a concentration of 1 ppm [5]).

Besides, only a half of the known dyes are regarded as biodegradable. The other half (about 53%–55%) is considered persistent in the environment [6], producing high amounts of textile dyes in water ecosystems with all the connected issues. Many textile dyes in fact are toxic, mutagenic, carcinogenic, being also able to lower light penetration in water bodies and therefore jeopardizing photosynthetic activity [7,8,9]. The legislators faced such issue since a long time. For instance, the European directive 2002/61/EC forbids the use of some derivatives from azo dyes [10]. More generally, a sustainable management is required by the European Water Framework Directive (2000/60/EC [11]).

The textile industry, in addition to this, has one of the greatest water demands (up 200 L per kg of textiles fabricated [1,12]), usually met by high quality fresh water, whereas the re-use of wastewater is still not very common [1]. At the same time, all industrial sectors are increasing their water demands, and the water supplies are dwindling, resulting in higher water costs (up to almost 6 €/m^3^ in the European Community [1]) and stricter environmental policies and regulations [1,12].

Following these inputs, the scientific community is working hard to develop novel and economically sustainable methods for the detoxification of textile wastewaters, aiming to respect the prescribed quality criteria and thus close the water cycle. Many biological, physical, and chemical approaches have been proposed [5,6,7,12,13,14]. However, all these methods suffer from some drawbacks still preventing their wide and sustainable application. For instance, the common biological (both aerobic and anaerobic) activated sludge treatment is not very flexible when faced with the high variety in textile wastes composition (e.g., pH, conductibility, turbidity); the same chemical composition of dyes is usually very recalcitrant and can greatly differ among various industries, and in the same plant over different periods of time (Table 1) [12,15,16]. Even if significantly less expensive than other approaches, biological treatment commonly requires large areas, and not always allows satisfactory bleaching efficiency [7]. Chemical oxidation is not usually very efficient, and some methods (e.g., ozonation) suffer from unaffordable costs [12,17]. Adsorption and coagulation techniques usually lead to the production of more secondary wastes, whose disposal poses even greater economic and environmental concerns [18]. Photocatalytic decolorization is costly and involves UV light [6,19].

An alternative methodology is based on metalloporphyrins as effective oxidation catalysts, whose ability to degrade a wide range of substrates under mild operational conditions is well known [25,26,27,28,29,30,31,32,33,34]. Accordingly, in this review, we describe their use as efficient, biomimetic and possibly environmentally friendly alternative for the treatment of textile wastes.

## 2. Synthetic Metalloporphyrins

### 2.1. Redox Metalloporphyrins: An Overview

**Scheme 1:**
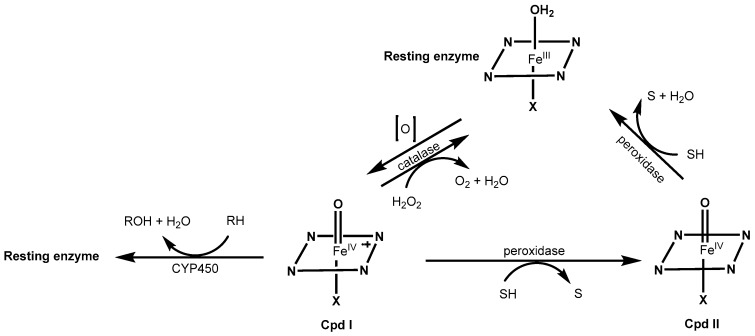
Biological oxidations involving heme proteins. Adapted from references [43,44].

Tetrapyrrolic macrocycles such as porphyrins and analogs are widely distributed in Nature, playing essential roles in vital processes such as photosynthesis, respiration, oxygenation and electron transport [35,36,37]. The chemical and physical properties displayed by these macrocycles, including their synthetic analogs, are responsible for their success in a wide range of fields such as supramolecular chemistry, biomimetic models for photosynthesis, catalysis, electronic materials, sensors, and medicine [33,38,39,40,41,42]. A particularly attractive and successful research field of metalloporphyrins is related with the possibility of mimicking biological oxidations involving heme proteins such as peroxidases, catalases and cytochrome P450 enzymes (CYP450), and the redox chemistry of oxygen, as summarized in a simplified form in Scheme 1 [43,44].

**Scheme 2:**
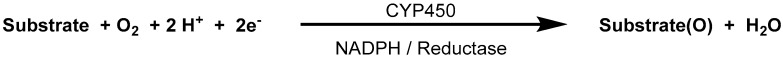
Cytochrome P450 reaction.

Catalases are responsible for the conversion of H_2_O_2_ into O_2_ and H_2_O through an intermediate of the type of Compound I (Cpd I). This intermediate and Compound II (Cpd II) are the two intermediates involved in the peroxidase action responsible by the one-electron oxidation of a large number of organic substrates. Cytochrome P450 enzymes are able to catalyze different types of reactions, namely the transfer of an oxygen atom into various organic substrates after reductive activation of dioxygen. In this last process, the two electrons needed for the reductive activation of molecular oxygen are usually provided by NADPH or NADH via a reductase (Scheme 2).

These monooxygenases are largely distributed in the majority of the living species and are particularly important in mammals for the oxidative metabolism of endogenous (e.g., steroids, prostaglandins) and exogenous compounds [45,46]. These enzymes possess the Fe(III) complex of protoporphyrin IX as the active site, which is linked to the protein by a cysteinate residue [47].

The versatility of these heme-thiolate enzymes, which catalyze not only oxidations but also reductions, dehydrations and isomerizations, are associated with the ability of iron, the metal ion in the inner core of the macrocycle, to adopt different oxidation states [47]. These oxidation states depend on the biological environment, the presence or absence of reductases and dioxygen, and on the intrinsic reactivity of the substrate [47].

**Scheme 3:**
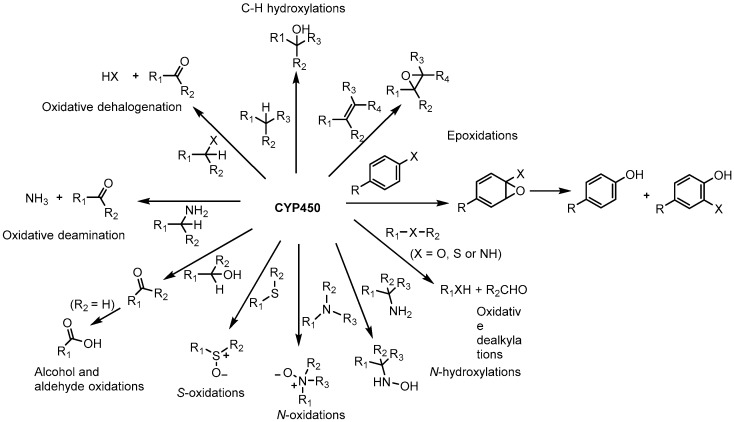
Main oxidation reaction pathways catalyzed by CYP450 enzymes. Adapted from references [44,47,48,49,50].

Some reactions catalyzed by the CYP450 enzymes involving the insertion of an oxygen atom are summarized in Scheme 3. These reactions can occur without bond cleavage (e.g., epoxidation, *N*- or *S*-oxidation) or with the cleavage of different type of bonds such as C–H, N–H, C–O or C–N. The cleavage of a carbon-heteroatom linkage can also happen when the C–H bond hydroxylation occurs at the α-position relatively to the heteroatom, such as in ethers, amines, amides and thioethers, leading to *O*-, *N*- or *S*-oxidative dealkylations [47,48,49,50]. However, this type of enzymes can also be involved in other oxidation processes, although without oxygen transfer, such as in the coupling of phenols and dehydrogenation reactions [47].

**Scheme 4:**
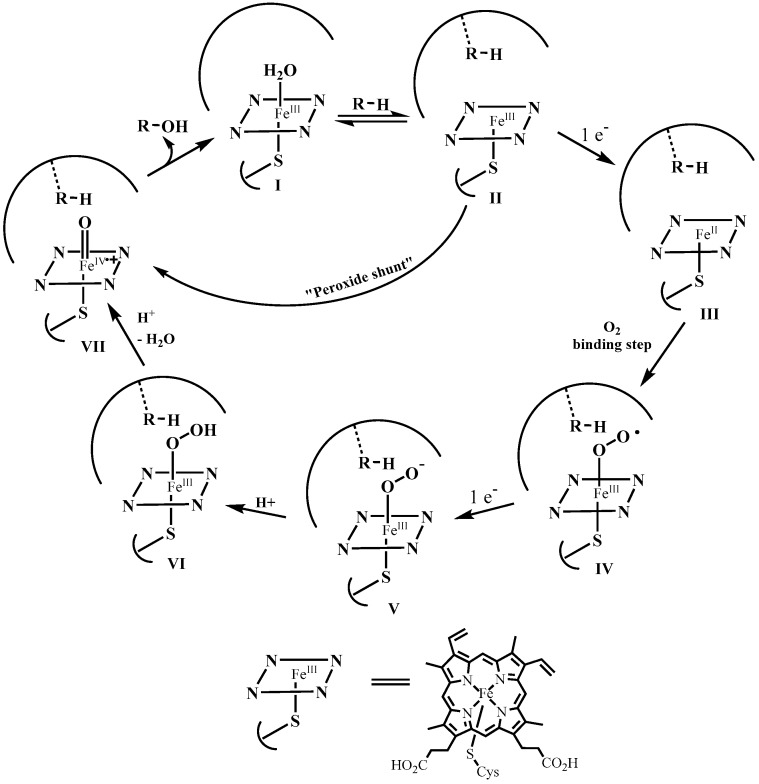
The catalytic cycle of CYP450 including the shorter “peroxide shunt”, and the structure of the Fe(III) complex of protoporphyrin IX. Adapted from [38,47,51,52,53,54].

As already mentioned above, the transfer of one oxygen atom into an organic substrate by CYP450 enzymes requires the reductive activation of dioxygen, the two electrons needed being provided in general by NADPH or NADH. The most accepted mechanism for this activation process is summarized in Scheme 4, and starts with the heme group in a six-coordinate low spin ferric state bearing water as the exchangeable distal ligand *trans* to the proximal cysteinate residue.

The axial water in the enzyme resting form **I** is lost after the binding of the substrate (RH), generating the five coordinate high-spin ferric complex **II**. The conversion of the ferric iron ion from low to high spin induces a change in the redox potential of the iron center from −330 to −173 mV after substrate binding [50,51]. This modification in the coordination sphere facilitates the one-electron reduction of the pentacoordinated ferric center, by an associated reductase with NADPH, generating iron(II) complex **III**. The binding of molecular oxygen to this pentacoordinated iron(II) leads to the superoxo iron(III) complex **IV**. These two entities can be responsible for some reactions catalyzed by CYP450 involving nucleophilic oxidant intermediates, such as the oxidative decarboxylation of aldehydes [51]. A second electron reduction allows the formation of a ferric peroxide adduct **V**, which can be protonated giving the hydroperoxide complex **VI**. Further protonation of **VI** leads to the heterolytic cleavage of the O–O bond with the loss of a water molecule to form a reactive ferryl-oxo porphyrin π-cation radical intermediate **VII**, which is comparable to Compound I (*Cpd I*) of peroxidase or catalase (Scheme 1). Finally, this intermediate **VII** transfers an oxygen atom to the substrate. Then, by product dissociation and coordination of water, complex **I** is regenerated [38,47,52,53,54]. More recently, another enzyme, structurally resembling both CYP450 and chloroperoxidase, has been found as a secreted glycoprotein in some fungi. The enzyme, which contains a ferriheme prosthetic group bound to the apoprotein by means of a thiolate from a specific cysteine residue, has been defined as peroxygenase (E.C. 1.11.2.1); it follows a catalytic cycle strictly paralleling the ”peroxide shunt” of CYP450 [55,56,57,58,59,60]. Therefore, no any external reducing agent (such as the expensive NAD(P)H) is required. Peroxygenases act on a wide substrate range, and therefore oxygenate (epoxidize and/or hydroxylate) aromatic rings, benzylic carbons, and even recalcitrant heterocycles such as pyridine. They can also act as bromoperoxidases, being on the contrary their chloroperoxidase activity quite negligible.

The discovery that some oxygen atom donors such as alkyl hydroperoxides, H_2_O_2_, peracids, sodium hypochlorite, sodium periodate, or iodosylbenzene can replace molecular oxygen and the two electrons needed in the normal cycle (Scheme 4), thus directly affording reactive intermediates **VI** or **VII** (“short circuit” or “peroxide shunt”; Scheme 4), is responsible for the high success of synthetic metalloporphyrins as catalysts in different oxidative processes.

Although nature has selected iron as the metal ion, extensive studies concerning the use of model systems to mimic the CYP450 role in biological systems showed that other metals, such as manganese, osmium, chromium, ruthenium, or cobalt are also able to generate very efficient catalytic systems [61,62,63,64,65]. These oxidation processes can have different practical aims, namely the possibility to: (i) obtain metabolites from drugs and other xenobiotics; (ii) lead from a drug to new potential drugs just in one step; (iii) obtain high-value compounds from readily available sources; (iv) obtain mutagens to in vitro studies; (v) degrade diverse pollutants released in the environment, such as drugs, pesticides, or textile dyes [61,62,63,64,65,66,67,68].

**Figure 1:**
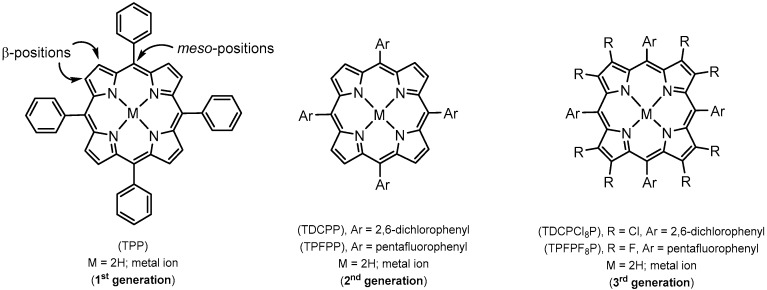
Structures illustrating the so-called first, second and third generation porphyrins [25].

Most of the successful studies concerning the use of synthetic metalloporphyrins (also known as metalloporphines, to underline the lack of organic substituents at the beta positions) in CYP450 biomimetic studies are based on 5,10,15,20-tetraarylporphyrins (also called *meso*-tetraarylporphyrins) and started in 1979 when Groves and coworkers reported that the iron(III) complex of 5,10,15,20-tetraphenylporphyrin (TPP) (Figure 1) was able to catalyze the oxidation of cyclohexane into cyclohexanol, and cyclohexene to the corresponding epoxide with iodosylbenzene as the oxygen donor [69]. In this landmark work, although the concept was proved, the macrocycle core was rapidly destroyed under the oxidative conditions of the reaction medium and affording also catalytically inactive µ-oxo complexes. The limitation of this so-called first generation of porphyrin catalysts was responsible for the appearance of the so-called second generation of porphyrin catalysts, bearing bulky groups with adequate electronic features in the aryl substituents, as exemplified in Figure 1 with some of the most used and efficient metalloporphyrins based on the 5,10,15,20-tetrakis(2,6-dichlorophenyl)porphyrin (TDCPP) and the 5,10,15,20-tetrakis(pentafluorophenyl)porphyrin (TPFPP) free-bases. Meanwhile, many groups all over the world decided to refine the structures of those catalysts by protecting also the β-pyrrolic positions of the macrocycle core, with halogens or nitro groups, in order to obtain even more robust and efficient catalysts, as shown in Figure 1 for the general structure of the so-called third generation of porphyrin catalysts, exemplified here with the β-octachloro-5,10,15,20-tetrakis(2,6-dichlorophenyl)porphyrin (TDCPCl_8_P) and β-octafluoro-5,10,15,20-tetrakis(pentafluorophenyl)porphyrin (TPFPF_8_P) free-bases [25].

**Scheme 5:**
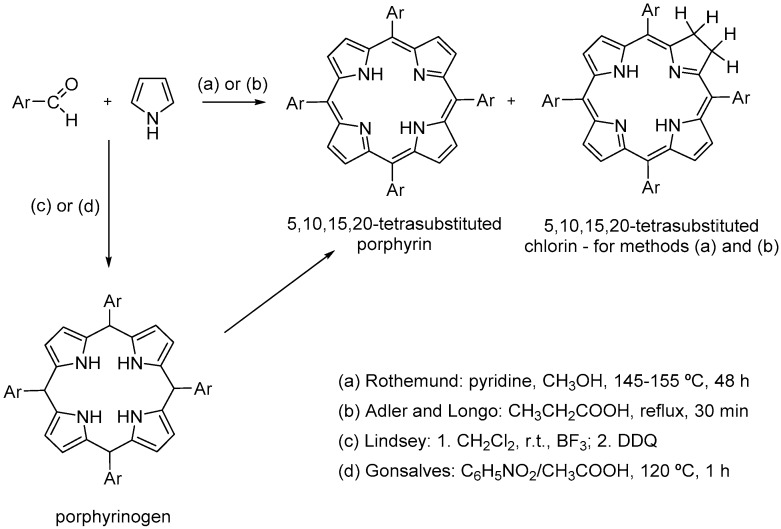
The synthetic methods usually employed to obtain 5,10,15,20-tetrasubstituted porphyrins from the condensation of different aldehydes with pyrrole.

Most of the porphyrinic ligands used in heterogeneous catalysis are based on the 5,10,15,20-tetraarylporphyrins of the first and second generation bearing the adequate functionalities that allow its immobilization on the support. In general, the access to the first and second generation of the free-base 5,10,15,20-tetraarylporphyrins is based on synthetic improvements of the landmark Rothemund reaction. In 1936, Rothemund was able to obtain, for the first time, a series of 5,10,15,20-tetrasubstituted porphyrins from the condensation of aliphatic, aromatic and heterocyclic aldehydes with pyrrole using a mixture of pyridine and methanol, at 145–155 °C in a sealed tube under anaerobic conditions (Scheme 5) [70,71,72]. The low yields reported and the contamination by the corresponding chlorin, identified by Calvin and coworkers [73,74], were the major drawbacks which were partially solved by the aerobic acidic conditions (propionic or acetic acid) described later by Adler and Longo [75]. Although under these acidic conditions important improvements in the porphyrin yields were reported, the presence of chlorin was still a limitation. The elimination of this contamination required the posterior treatment of the reaction mixture with 2,3-dichloro-5,6-dicyanobenzoquinone (DDQ) [76] or, as reported by Gonsalves and coworkers [77], by doing the condensation in a mixture of nitrobenzene and acetic acid. The obtainment, in some cases, of the porphyrin by simple crystallization directly from the reaction medium is also a friendly advantage of the oxidative mixture reported by the Portuguese group.

An important contribution, allowing obtaining porphyrins from aldehydes that could not support the Adler refluxing acidic conditions, was reported by Gonsalves and Lindsey in the late eighties, by using a two-step synthetic strategy (Scheme 5). The tetramerization of the monomeric units using chlorinated solvents was performed in the presence of trifluoroacetic acid (TFA) or BF_3_ at room temperature, in the absence of oxygen and light in order to maximize the formation of the porphyrinogen, followed by the oxidative step with DDQ or *p*-chloranil. Gonsalves and Pereira used this strategy to prepare 5,10,15,20-tetraalkylporphyrins [78], and Lindsey to prepare 5,10,15,20-tetraarylporphyrins with yields between 30% and 40% [79,80]. More recently, Sharghi and Nejad reported an efficient synthetic access to several 5,10,15,20-tetraarylporphyrins (yields ranging between 20% and 65%) by condensation of pyrrole and aryl aldehydes at room temperature and using equimolar amount of PCl_5_ or CF_3_SO_2_Cl in the presence of air as an oxidant [81,82].

The possibility to apply microwave irradiation in the preparation of a series of 5,10,15,20-tetraarylporphyrins is being considered by different authors and, in most of these studies, the presence of an acid was considered essential [83,84,85,86,87]. Interestingly, Pereira and coworkers reported that the 5,10,15,20-tetraaryl and 5,10,15,20-tetraalkylporphyrins can be obtained in good yields under microwave irradiation using water, which acts simultaneously as solvent and acid catalyst [88].

In some cases, the starting aldehyde has not the adequate functionality to be grafted into the support, requiring extra functionalization such as the introduction of, e.g., sulfonic or nitro groups that can be further reduced to amino groups. The access to the third generation of porphyrin catalysts requires also posterior halogenation or nitration by using fuming nitric acid or a mixture of acetic anhydride and zinc nitrate [89,90,91]. The most widespread metalloporphyrins cited in this paper, and their respective acronyms are listed in Table 2.

### 2.2. Substrate Specificity

Since the confirmation that synthetic models based on porphyrins are able to mimic the action of heme thiolates, the type and number of substrates studied are countless. One of the important goals for developing efficient biomimetic models is related to the necessity of developing sustainable, environmentally benign, safe, and clean methodologies for synthetic organic chemistry [92,93,94,95,96,97,98]. In particular, the oxidation reactions needed to convert bulk chemicals (e.g., from petroleum products) into useful products are amongst the most problematic transformations in industrial core technologies (expensive, not selective, and frequently performed under environmentally malignant conditions), hence justifying the huge investment made by different research groups studying the oxidation of aliphatic (alkanes and alkenes) and aromatic hydrocarbons (e.g., toluene, ethylbenzene, and styrene) or phenolic derivatives under homogeneous and heterogeneous conditions [61,62,64,65,99,100,101,102,103,104]. This is also a challenging topic if considering the possibility to use natural and abundant substrates such as terpenes giving rise to high-value compounds with organoleptic or pharmacological interest. In particular, Meunier and coworkers had an important contribution to this field by studying the epoxidation of 3-carene with NaClO in the presence of Mn(III) porphyrins and the oxidation of α-pinene and α-terpinene with the same oxidant and KHSO_5_ [105,106]. The possibility to use H_2_O_2_ as oxidant and Mn(III) porphyrin catalysts of the second and third generation was developed by Cavaleiro and coworkers in the oxidation of 3-carene [107] and other terpenoids such as 1,8-cineole, methyl dehydroabietate [108], carvacrol, thymol, or *p*-cymene [109], as revised recently [33,65].

Considering the biological and pharmacological importance of steroids, several groups studied also readily available steroids aiming to obtain new bioactive compounds using simple biomimetic oxidative strategies. In fact, a considerable number of studies concerning the epoxidation and hydroxylation of readily available steroid derivatives by using different oxidants such as *tert*-butyl hydroperoxide (TBHP), 2,6-dichloropyridine *N*-oxide, iodosylbenzene or H_2_O_2_ as oxygen donors in the presence of Fe, Ru, Mn, or Os porphyrins were reported [110,111,112,113,114,115,116,117,118].

In general, the key route for the elimination of xenobiotics, such as drugs, after their introduction into the organism is initiated by oxidation reactions catalyzed by enzymes of the cytochrome P450 (CYP450) group. Therefore, the possibility to modulate their metabolic activation by biomimetic synthetic models has been also responsible to intensive studies all over the world. Most of the events catalyzed by CYP450 enzymes, namely aliphatic and aromatic hydroxylation, double bond epoxidation, *N*-oxidation, *N*-dealkylation, *S*-oxidation, decarboxylation can be efficiently simulated by biomimetic models under proper conditions [51,61,63,66,119]. This approach can give access to important metabolites in adequate amounts, thus facilitating the prediction studies of their useful or harmful effects.

Nowadays, the negative impact caused by the presence of organosulfur compounds such as the recalcitrant benzothiophenes (BTs) and dibenzothiophenes (DBTs) in fuels is well established [120]. In fact, the combustion of organosulfur compounds is usually associated with the formation of acid rains, the poisoning of the catalysts and the corrosion of the internal parts of combustion engines [121]. Considering the restrictive regulations limiting the sulfur content on petroleum products, allied to the technical limitations of the common hydrodesulphurization procedures, synthetic metalloporphyrins are also being considered in the research studies concerning the so-called oxidative desulfurization (ODS) methodologies [122,123]. In some of those studies the authors selected H_2_O_2_ as oxidant and manganese(III) or iron(III) complexes of the second generation, affording the corresponding fully oxidized derivatives with high conversions at room temperature. So, the high potential of the metalloporphyrin complexes as catalysts for the sulfoxidation of the *S*-recalcitrant benzothiophenes and dibenzothiophenes by H_2_O_2_ was demonstrated [122,123].

Polluting phenolics and humic substances have been also suggested as substrates for synthetic metalloporphyrins, in an intriguing series of papers dealing with the development of processes for the treatment of polluted soils [124,125,126,127,128]. Dehalogenation of halogenated phenols, mimicking chloroperoxidases has been also described [129,130,131,132,133].

In recent years a particular attention was also given to the use of biomimetic models in the oxidative degradation of lignin [134]. This subject is also considered a hot topic since this renewable polymer can be an excellent source of aromatic compounds of low molecular weight. Additionally, the selective degradation of lignin and its removal from the carbohydrate component of wood is a key step in pulp and paper industry [29,135].

Another important field of research where the oxidative potential of these biomimetic models is being considered is related with the presence of drugs and its metabolites, pesticides, or dyes in natural waters, being a serious problem to aquatic and human life. In particular, textile dyes, due to their chemical inertness, toxicity, and high production, are of special environmental concern and the potential of metalloporphyrins to degrade different dyes will be the subject of a more profound discussion in the next section.

### 2.3. Immobilization and Emulation of Ligninolytic Enzymes

A huge amount of papers reports the catalytic activity of metalloporphyrins in homogenous phase [33,127,136,137,138,139], even in the context of the treatment of textile dyes [140,141]. However, the heterogenization of the catalyst is a striking priority for the feasibility of a large scale process [29,142]. Several factors prompt such a requirement:

- The synthesis of metalloporphyrins is quite expensive, and at the same time, a significant part of their initial catalytic activity is retained at the end of the process [143,144]. Accordingly, their recovery is significantly cost-effective;
- Toxicology of metalloporphyrins is still almost unknown. So, a separation from reaction mixture is usually required, further increasing the cost of the process [29];
- If performed correctly, i.e., emulating ligninolytic peroxidases active site [28,145], immobilization could increase stability and efficiency of the catalyst [25];
- Metalloporphyrins are intensely colored, as well as the dye substrates, frustrating the whole purpose of the process when used in free form to bleach dyes.

Immobilization, however, poses also some potential drawbacks in addition to all these positive effects. For instance, too strict confinement of catalysts inside a narrow 3D network of the support could lead to some mass transfer limitations, or at least sterical hindrance of immobilized catalysts. Especially, if the immobilization is not performed properly (i.e., not proper size of support pores, excessive crowding of catalysts onto the surface of the support).

Several approaches for metalloporphyrins heterogenization have been proposed, somewhat resembling the methods used for protein immobilization [146,147,148]. The main methods are encompassed in Table 3.

Adsorption is the most common method for immobilization of metalloporphyrins, not involving any grafting of supports or elaborate coupling reactions. Adsorption relies on a combination of overlapping non-specific weak interactions [146], being not always easily distinguishable from other techniques, such as ion exchange.

Physical inclusion (i.e., encapsulation, and entrapment) is also possible, involving any interaction between catalyst and support, but the first is simply included in the tridimensional frame of the later. Therefore, no chemical modification of catalyst occurs, allowing theoretically the retention of its whole catalytic activity.

The strongest interaction possible can be achieved, on the contrary, through covalent bonds. Consequently, this approach tops the others about the minimization of leakage issues. Several reactions have been developed to perform the immobilization of the most diffused metalloporphyrins (particularly, the second generation ones, Figure 1).

**Scheme 6:**
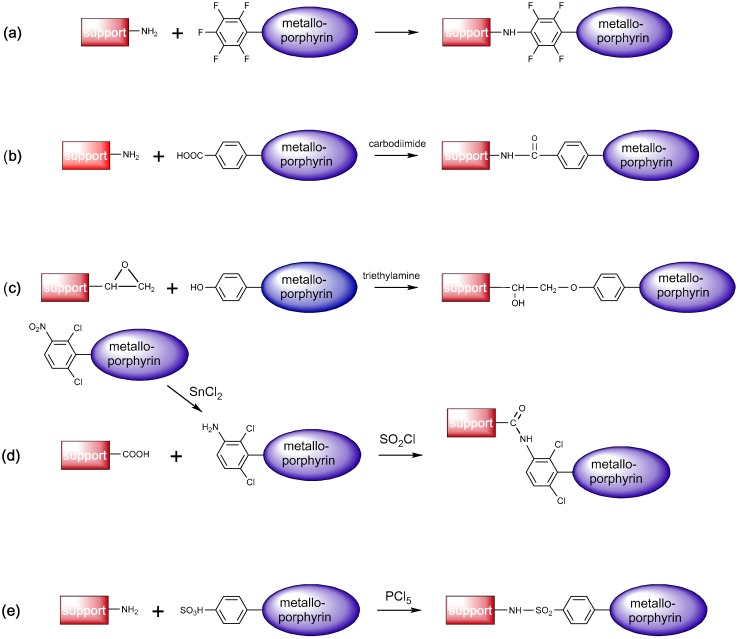
Some of the most common methods for the covalent immobilization of metalloporphyrins.

Perfluorinated porphyrins, such as the Fe(III) and Mn(III) complexes of 5,10,15,20-tetrakis(pentafluorophenyl)porphyrin TPFP can be for instance immobilized by nucleophilic addition from aminofunctionalized support, yielding a stable secondary amine [68,103,149], as shown on the path (a) of Scheme 6).

Carboxyfunctionalized porphyrins, such as the Mn(III) complex of 5,10,15,20-tetrakis(4-carboxyphenyl)porphyrin, can be activated through a carbodiimide [146] to give amide bond with aminopropylated supports [150] (path (b) Scheme 6). In the case of methoxylated analog porphyrins, such as the Mn(III) complex of 5,10,15,20-tetrakis(4-methoxycarbonylphenyl)porphyrin, a similar reaction occurs in the presence of NaH as the condensation activator [151].

Hydroxyl porphyrins, such as the Mn(III) complex of 5,10,15,20-tetrakis(4-hydroxyphenyl)porphyrin, can be easily immobilized on epoxyfunctionalized supports. The presence of suitable basic catalysts (such as triethylamine, NaOH, or Na_2_CO_3_) affords the formation of a stable alkyl-aryl-ether bond [152], as shown in the path (c) of Scheme 6.

Covalent immobilization of dichlorophenylporphyrins, such as the Mn(III) complex of 5,10,15,20-tetrakis(2,6-dichlorophenyl)porphyrin, TDCP, is not direct, usually requiring the insertion in the *meso*-aromatic ring of –NH_2_ functions. These aromatic amines can yield in turn amide bond with carboxyl-grafted supports, upon thionyl chloride activation [153], as depicted in the path (d) of Scheme 6.

Thionyl chloride (or other activating halides like PCl_5_ [29]) can be used as well to activate sulfonated porphyrins, such as the Mn(III) and Fe(III) complexes of 5,10,15,20-tetrakis(2,6-dichloro-3-sulfonatophenyl)porphyrin, yielding sulfonamide bonds with aminated supports [153] (path (e) Scheme 6).

Each technique, however, shows some drawbacks, as summarized in Table 2. Physical inclusion, adsorption, and ion exchange immobilization, in fact, suffer from too weak linkage between metalloporphyrin and support, involving a possible leakage of the catalyst during reaction. This circumstance should be avoided by all means, since it poses a double economical concern. In fact, a part of still active catalysts is relentlessly loss. On the other hand, reaction products could be seriously contaminated by the colored metalloporphyrin.

Conversely, covalent immobilization provides a strong interaction, minimizing leakage issues. But during the immobilization reactions chemical modification of the catalyst is possible. All these approaches have found, in any case, extensive applications for the immobilized metalloporphyrins [32,152,154,155,156,157].

However, none of these approaches strictly emulates ligninolytic peroxidase active site. In fact, in lignin peroxidase (LiP), manganese peroxidase (MnP), and versatile peroxidase (VP), the imidazole from a histidine side chain coordinates iron(III)-heme. Similarly, the thiolate function from a cysteine residue is responsible for immobilization of the prosthetic group in peroxygenases and in some cytochromes [29]. Besides, the favorable effect of axial ligand on metalloporphyrins stability and efficiency has been clearly demonstrated [25,29,104,158,159].

**Figure 2:**
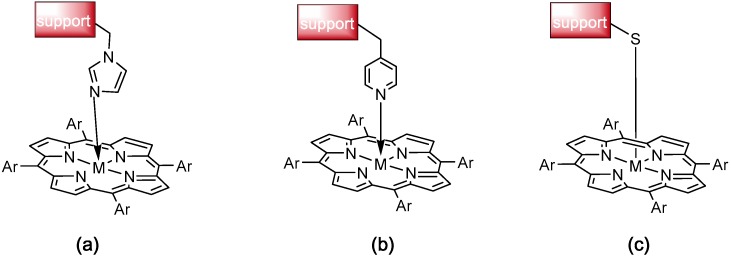
Immobilization of metalloporphyrins through coordination bond. The most common ligands are imidazole (**a**) to emulate the active site of ligninolytic peroxidases [28,143]; pyridine (**b**) [26,31]; and mercapto function (**c**) to emulate the active site of cytochrome P450 and peroxygenase [30].

Accordingly the immobilization through coordinative interaction onto imidazole coated supports has been also proposed [143,160,161,162] (Figure 2a), avoiding the need of adding imidazole as the co-catalyst to the reaction mixture [163,164,165].

Somewhat paralleling the emulation of ligninolytic peroxidases active site, also immobilization through coordinative bonds onto pyridine- [26,27,31] (Figure 2b), and thiol-grafted supports [30] (Figure 2c) has been reported with promising results both in terms of metalloporphyrin loading onto the supports and catalytic performance of the adduct. Besides, the effect of different ligands on specificity, selectivity, and promotion of different catalytic paths (in particular one-electron oxidations versus direct oxygenation [29]) has been clearly demonstrated [26,30,104].

## 3. Textile Dyes

Dyes are chemical compounds characterized by absorbing in the visible region of the electromagnetic spectrum (400 to 700 nm). Until the XIXth century, most dyes came from natural sources such as leaves, roots, berries, insects, molluscs, or were mineral substances.

The Perkin reaction opened the way to the development of a huge number of synthetic dyes, belonging to different classes, according to the structural motifs representing the chromophoric moieties within their chemical structures [166].

Industrial dyes represent a large group of organic compounds that could have undesirable effects on the environment [167] and, in addition, many of them can pose risks to human health [168,169,170]. By definition, an industrially applicable dye should be a quite resistant compound, capable of surviving against harsh conditions such as prolonged exposition to sunlight, and harsh washing and/or bleaching treatments. Accordingly, they are very often recalcitrant with respect to chemical and biological degradation processes [169,171,172,173].

Unfortunately, nowadays the demand has led to the widespread use of synthetic colors. They represent the most economical solution for industries, for both the higher performances compared to the natural ones, and for considerably lower costs.

The synthetic dyes have to show the two features of being absorbed by the fabrics to be dyed (affinity) and of remaining fixed (solidity).

These dyes are very often aromatic compounds with largely delocalized π electrons, responsible for the absorption of visible light. In particular, the dye moiety, primarily responsible for the coloration of the compound, is defined as the chromophore. Color intensity is reinforced by the presence of substituents, capable of participating to the electron delocalization, and called auxochromes. These are often involved in other important properties, such as the solubility of the dye in water (or sometimes in non-aqueous solvents) as well as its affinity and solidity [166,174].

**Figure 3:**
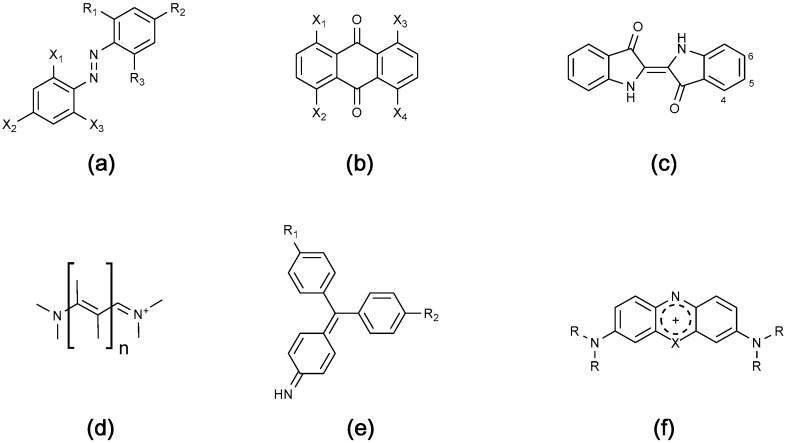
Core structure of some of the most used textile dyes. (**a**) azo; (**b**) anthraquinone; (**c**) indigo; (**d**) cationic; (**e**) triphenylmethane; (**f**) other cationic dyes with condensed tricyclic systems derived from acridine/phenazine/phenoxazine or similar structures, where one heteroatom bears a net positive charge.

For the purposes of the present review, industrial dyes could be conveniently classified by following a chemical/structural criterion [169], rather than a technical one. So, the main classes of industrial dyes, that have been the target of biomimetic oxidative degradation studies, under metalloporphyrin catalysis, are briefly described below:

*Azo Dyes*. These synthetic dyes are typical for the presence of one or sometimes more chromophoric groups –N=N–. The azo bridge joins two aromatic rings, one of which bears electron-releasing, and the other electron-withdrawing substituents (Figure 3a. R_1_, R_2_, R_3_ represent hydrogen and/or electron-releasing substituents such as –OH, –OCH_3_, –NH_2_; X_1_, X_2_, X_3_ represent hydrogen and/or electron-withdrawing groups such as –NO_2_ and –SO_3_^−^).

The aromatic rings could virtually derive from benzene, naphthalene, or also heteroaromatics. Commercially, azo dyes represent the most important class of synthetic dyes, as they show at the same time facile production at low cost, bright colors with a number of beautiful different nuances, high affinity and solidity. The –N=N– moiety, placed between the two aromatic portions, is called azo group and it is a strong chromophore conferring a bright color, usually ranging from yellow to orange to red [175]. An example of an azo dye is Methyl Orange, used for dyeing wool and silk, and perhaps better-known by far as a popular pH indicator.

As a general feature, azo dyes are toxic to many forms of life. Many have been withdrawn from the market—in particular among those used for coloring food—because of their carcinogenic properties. Their toxicity arises from their in vivo reduction to aromatic amines upon enzymatic cleavage of the azo group [170,176,177]. Therefore, their massive presence in wastewaters, released by textile plants, represents a major concern in the perspective of solving the severe pollution problems caused by such plants.

*Anthraquinone dyes*. These derive from the almost colorless 9,10-anthraquinone. The industrially relevant derivatives contain powerful electron-donating groups such as amino or hydroxy substituents as auxochromes, in at least one of the four α positions (Figure 3b, X_1_, X_2_, X_3_, X_4_ represent electron-releasing substituents such as –OH and –NH_2_, which can also form hydrogen bonds with the adjacent carbonyls. Up to three substituents could be hydrogen atoms; additional substituents could be present at the β positions). Some hydroxyanthraquinones are of natural origin, but can be industrially synthesized with sharply lower costs, whereas many others (amino-, nitro-, and/or sulfo-derivatives) are synthetic. Simple anthraquinone dyes include Alizarin (1,2-dihydroxy-anthraquinone), once obtained from *Rubia tinctorum*, and its non-natural derivative Alizarin Red S (1,2-dihydroxyanthraquinone-3-sulfonic acid, ARS). Whether natural or based on natural molecules, anthraquinone dyes show cumulative toxicity [178]. Anthraquinone dyes are among the most durable industrial dyes and therefore are used when high resistance against sunlight and/or harsh environmental conditions is required. Such a feature could obviously be a significant problem for their degradation [144]. Unnatural, α-amino-substituted hydroxyanthraquinones are valuable dyes whose colors range from green to blue to purple.

*Indigoid dyes*. These are named after indigo (Figure 3c) obtained from the plant *Indigofera tinctoria* [179]. One or more positions on each benzene ring could bear different substituents in both natural and synthetic indigoids. Another prominent dye of this class is 6,6′-dibromoindigo, which is the Tyrian purple, produced as a colorless precursor by the shellfish *Murex brandaris* [180]. Natural indigoids are insoluble pigments rather than dyes, but can be transiently solubilized by reduction, and in turn directly fixed to the fibers upon re-oxidation by air (vat dyes). Indigoids are typical for their exceptional resistance to sunlight, durability and solidity.

*Cationic dyes*. Cationic dyes should be defined as dyes whose positive charge(s) are an essential part of their chromophores [181]. They should not be confused with other dyes, whose positive charges are not involved in the chromophoric structures, but have the main function of conferring solubility in water and/or the ability of forming strong ionic interaction with negatively charged fibers. Several applications have been described for these dyes [182,183]. In a stricto sensu cationic dyes belong to the cyanine group (in turn a subclass of the polymethine family, Figure 3d), showing a variable number of conjugated double bonds ending with two nitrogen atoms at the two termini of the unsaturated chain. The odd number of carbon atoms forming the unsaturated chain implies the presence of a net positive charge, delocalized between the two nitrogen atoms at the ends of the polyene bridge. Many dyes belong to the group, although many are conventionally classified as diphenylmethane, triphenylmethane, fuchsoneimine and benzophenoneimine derivatives (Figure 3e, showing as an example the fundamental structure of fuchsoneimine, where R_1_ and R_2_ represent generic amine substituents) and moreover their condensed and/or internally bridged derivatives [184].

Other cationic dyes contain condensed tricyclic systems derived from acridine, phenazine, phenoxazine, phenothiazine, xanthene, where one heteroatom bears a net positive charge (Figure 3f, showing a generic azine); the X atom can be O, S, N. The positive charge on the ring heteroatom is partially delocalized on the two amino substituents). Such systems could be regarded as azomethine isologs where the nitrogen atom could be eventually changed in an oxygen or sulfur atom, in the form of an oxonium or sulfonium cation, respectively.

## 4. Application of Metalloporphyrins in the Decolorization of Textile Dyes

### 4.1. Biomimetic Bleaching of Industrial Dyes

As previously underlined (Section 3), industrial (more particularly, textile) dyes are organic molecules, almost invariably containing heteroatoms such as oxygen, nitrogen, sulfur, and combinations of these. As such, they are potentially oxidizable by a variety of different oxidizing agents, ranging from molecular oxygen to more active and efficient compounds such as hydrogen peroxide, some alkyl- or acyl-peroxides, peroxyacids, possibly in the presence of suitable catalysts. Sadly, the most active oxidizing agents are very often unstable species, could in turn release exhaust products causing environmental and toxicological concerns, and are unacceptably expensive. In this sense, hydrogen peroxide represents a good compromise between cost and effectiveness [28,185]:

- It is reasonably stable and safe for production, transport, storage, delivery, and usage.
- Its relatively low price has allowed a more and more wide use in many technological applications.
- Last but not least, side products arising from its action are only water and molecular oxygen, causing no environmental and toxicological concern.

Hydrogen peroxide is much more reactive than molecular oxygen, due to its substantially lower activation energy. Although the redox potential for H_2_O_2_ reduction is higher at acidic pH values, where it acts as a weak electrophile toward electron-rich substrates, as a rule it is generally much more effective as an oxidant for organic compounds in alkaline environment. Under such conditions, the concentration of the hydroperoxide ion HOO^−^ becomes important, which explains the reactivity towards electrophilic positions of the oxidizable substrates. In the pH range 10–12, hydroperoxide anion can react with non-dissociated peroxide [186], giving rise to the very strong oxidizer hydroxyl radical, and superoxide anion. The latter acts as a relatively weak oxidizer [187], preferentially attacking electrophilic places (when present) in the oxidizable organic molecules, or can also react further with each other releasing molecular oxygen. Moreover, two superoxide anions can produce the very aggressive singlet oxygen. Therefore, within that pH range hydrogen peroxide can indirectly oxidize and bleach substances, which are quite resistant to the same oxidizer when pH is lower than 10. However, a substantial peroxide fraction is wasted as molecular oxygen, so the overall efficiency of the oxidation process can be noticeably lowered.

The above described picture can dramatically change in the presence of a number of catalysts, such as polyoxometalates (POMs), which have been very recently reviewed [188]. Many POMs are effective oxidation catalysts, and some can profitably use hydrogen peroxide as the oxidizer. However, despite of their efficiency and stability, that allow catalyst recycling, their solubility in the (aqueous) media poses a serious concern for their recovery, to avoid unwanted losses and dispersion of heavy metal compounds in treated wastewaters.

As noted in Section 2.3, redox-active metalloporphyrins usually behave as more or less efficient oxidation catalysts. For the particular purpose of textile plant wastewater bleaching, they have to be immobilized, to allow their retention within the reactor, so determining substantial savings (they are rather expensive compounds) and preventing pollution and toxicological concerns. However, a number of studies have been carried out also with metalloporphyrins in solution, that have brought a substantial contribute in elucidating degradation/bleaching mechanism(s) for some classes of industrial dyes. To a certain extent, metalloporphyrin catalysts could be compared to high-redox-potential peroxidases [189], which are on the whole much more active, but also substantially costlier.

Somewhat surprisingly, no degradation studies are reported on indigoid dyes, under metalloporphyrin catalysis, although water-soluble indigo carmine is a good substrate for peroxidases (*vide infra*). Therefore, the efficient, biomimetic oxidative bleaching of indigoids by means of redox-active metalloporphyrins looks highly likely.

The applications to azo, anthraquinone and cationic dyes are reported in Section 4.1.1, Section 4.1.2 and Section 4.1.3, respectively. Finally, applications of the same catalysts to dyes of different classes are reported in Section 4.1.4.

#### 4.1.1. Azo Dyes

**Figure 4:**
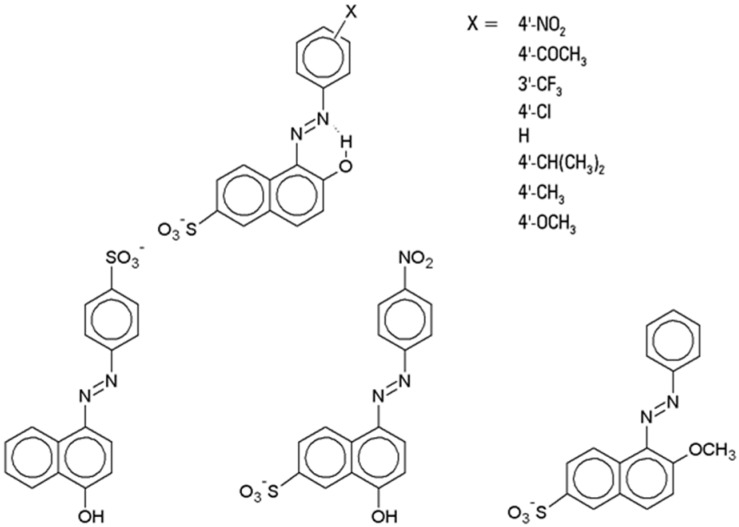
Substituted phenylazo derivatives of substituted 1- or 2-naphthols studied as substrates of the iron(III) complex of 5,10,15,20-tetrakis(2,6-dichloro-3-sulfonatophenyl)porphyrin (TDCSP).

Some synthetic iron porphyrins have been tested as catalysts for azo dye bleaching. Hodges and colleagues [190,191] have prepared the relatively stable *Cpd II* analog of a sterically hindered, anionic, water-soluble iron(III) complex of 5,10,15,20-tetrakis(2,6-dichloro-3-sulfonatophenyl)porphyrin, and assessed its effectiveness in bleaching some azo dyes under different working conditions. The chosen dyes were all phenylazo derivatives of substituted 1- or 2-naphthols (Figure 4). As such, these azo dyes exist in tautomeric equilibrium with the corresponding hydrazones. The studied pH range was between 6.93 and 12.68, which influenced the protonation state of the 2-naphthol derived dyes, where a strong internal hydrogen bridge exists between the phenolic hydroxyl and the distal nitrogen of the azo group. Therefore, the acidity of the phenolic function is reduced, and under neutral conditions each dye effectively exists as a mixture of the azo and hydrazone forms (each with its own reactivity towards the oxidizing agent, and with a prevalence of the hydrazone form over the conventional azo form), whereas at alkaline pH values such tautomerism is abolished as the dyes largely exist as the corresponding delocalized anions. On the whole, the *Cpd II* analog of the studied iron porphyrin behaved as an efficient oxidant towards this series of azo dyes.

**Scheme 7:**
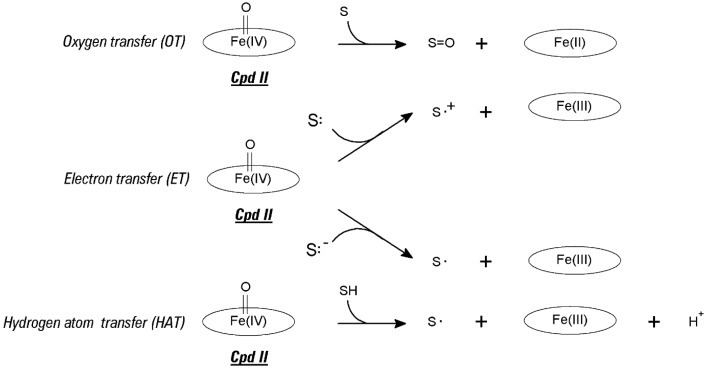
The reductions of metalloporphyrin *Cpd II* analog can follow three main pathways: oxygen transfer (OT), hydrogen atom transfer (HAT), and electron transfer (ET) (S = substrate). OT path under these conditions was not observed.

In principle, three different mechanisms can operate in oxidation by the *Cpd* *II* analog, the studied iron porphyrin: (i) oxygen transfer (OT); (ii) electron transfer (ET); and (iii) hydrogen atom transfer (HAT). The general mechanisms are encompassed in Scheme 7. Of these, the OT has been ruled out for that iron porphyrin in aqueous environment. ET was the only possible mechanism under alkaline conditions, where the studied dyes exist as the corresponding anions. Also the HAT mechanism seems to be unlikely, as the authors noted that when methoxyl group was substituted for hydroxyl group in position 2 of the naphthalene ring, only a marginal difference in the reaction rate was observed.

When the same ferriporphyrin was treated with a stoichiometric excess of peroxyacids such as *meta*-chloroperoxybenzoic acid or magnesium monoperoxyphthalate, the *Cpd I* analog is the first reaction product. This can quickly attack the azo dye with a two-electron mechanism, therefore restoring the resting ferriporphyrin, or alternatively can comproportionate with the resting ferriporphyrin, thus giving rise to two molecules of *Cpd II* analog.

**Scheme 8:**
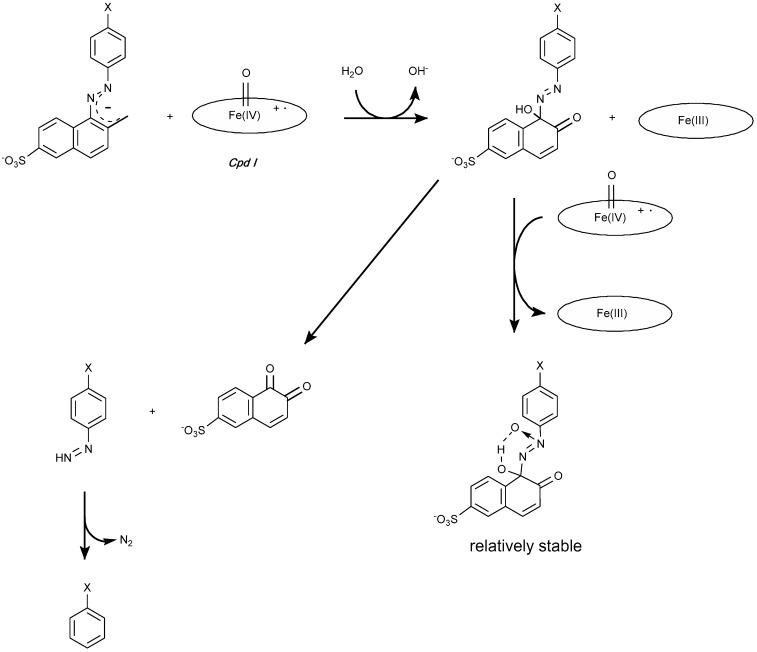
At alkaline pH (almost 12) *Cpd I* analog converts 1-phenylazo-2-naphthol dyes in 1-carbinols. These in turn can be cleaved unsymmetrically by *Cpd II* (X = –NO_2_ or –OCH_3_).

The latter slowly reacts with the dye, following a one-electron oxidation mechanism that again leads to the resting ferriporphyrin. Interestingly, when the peroxyacid concentration is increased far beyond the stoichiometric requirements, the overall oxidant accountability tends to decline. This has been attributed to the competing comproportionation reaction, consuming the very active *Cpd I* analog and therefore slowing the dye bleaching. At sharply alkaline pH (11.88) 1-phenylazo-2-naphthol dyes mainly exist as the corresponding anions, which are attacked by the *Cpd I* analog by a direct oxygen transfer (or by an oxygen rebound mechanism [192]), giving rise to 1-carbinols. Upon hydrolysis, such carbinols are cleaved unsymmetrically, giving rise to *o*-naphthoquinone derivatives (and their degradation products) and phenylazenes (and their degradation products), Scheme 8.

**Scheme 9:**
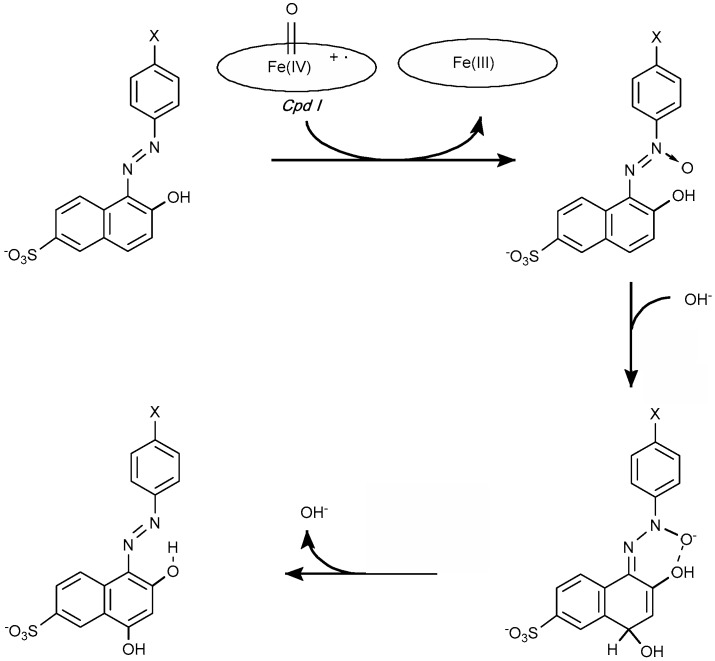
At pH 9.30 a nucleophilic attack of a hydroxide ion at position 4 of the naphthol ring takes place after the reaction with *Cpd I* analog. No cleavage of azo moiety was detected, whereas Wallach rearrangement occurs.

At pH 9.30, the same dyes are largely un-ionized, with the hydrazone tautomers prevailing. Under these conditions, transient β-azoxycompounds arise, which in turn undergo a nucleophilic attack of a hydroxide ion at the position 4 of the naphthol ring, whereas the original azo moiety is restored as it happens along the Wallach rearrangement, Scheme 9.

**Scheme 10:**
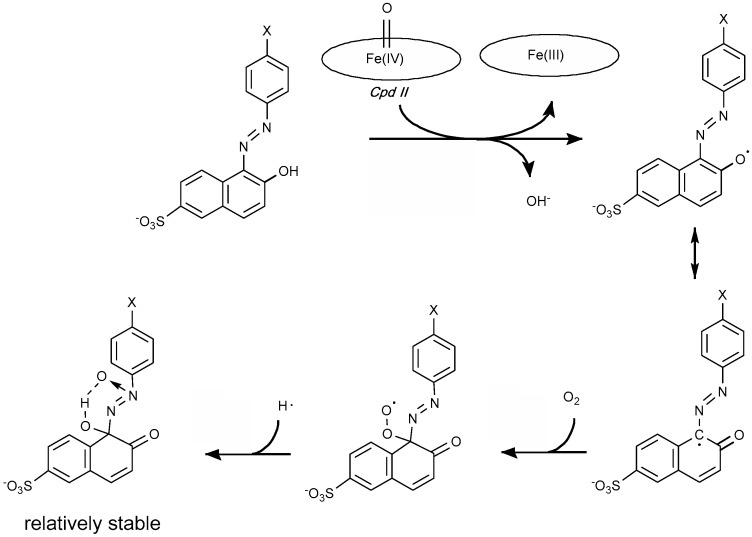
At neutral pH, the oxidizing species was *Cpd II* analog through HAT transfer. The arising radical from the azo dye can in turn react with molecular oxygen, yielding the same product observed at alkaline pH.

Finally, when pH was fixed at 6.93 and the oxygen donor was *tert*-butylhydroperoxide, *Cpd II* analog directly arose together with ^t^Bu–O∙. Both are unable to perform direct (or rebound) oxygen transfer, and act by a HAT mechanism on the 2-naphthol function of the dye. The arising free radical can react with molecular oxygen, and further transformations afforded the same product obtained at pH 11.88 with peroxyacids, Scheme 10.

With concern to the 4-hydroxy isomers, in these compounds any intramolecular H-bonding between the naphthol hydroxyl and the azo moiety cannot take place, and therefore these compounds are distinctly more acidic than their 2-isomers are. Therefore, they largely exist as the corresponding anions also at lower pH values, and show lower oxidation potentials for the same reason [190]. The observation that the 1-naphthol dyes are more readily bleached in the presence of the studied ferriporphyrin catalyst in comparison with their 2-naphthol counterparts, when both the isomeric series are in their unionized state, strongly speaks in favor of a HAT mechanism under those experimental conditions. Such behavior strictly parallels that observed for the same dyes, oxidized by hydrogen peroxide in the presence of horseradish peroxidase or fungal ligninase [193].

The action of H_2_O_2_ on Acid Orange 7 in the presence of the Mn(III) complex of 5,10,15,20-tetrakis(4-carboxyphenyl)porphyrin, covalently immobilized onto Fe_3_O_4_ magnetic nanoparticles was studied [194]. Although no mechanisms for dye degradation were presented, the study underlined the high catalytic performances of the immobilized metalloporphyrin and its stability after repeated oxidation cycles.

**Figure 5:**
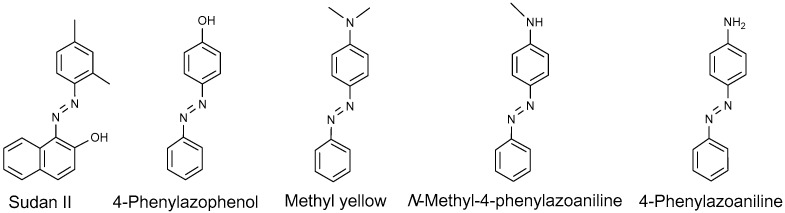
Several substituted azobenzenes have been proved to be substrates of Fe(TDCPP)Cl/MCPBA system.

In another study some amino-substituted azo dyes were studied to assess their degradation by *meta*-chloroperoxybenzoic acid (MCPBA) and iodosylbenzene, in the presence of the commercial Fe(TDCCP)Cl and Fe(TPFPP)Cl [195]. In particular, the Fe(TDCPP)Cl/MCPBA system oxidized a variety of substituted azobenzenes such as Sudan II, 4-phenylazophenol, Methyl Yellow, *N*-methyl-4-phenylazoaniline and 4-phenylazoaniline (Figure 5).

**Scheme 11:**
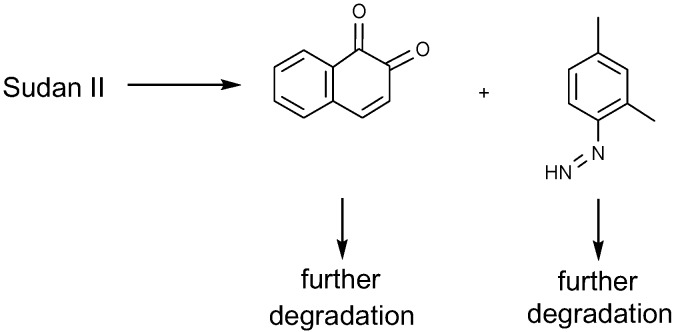
Sudan II was bleached in the presence of the Fe(TDCPP)Cl/MCPBA system with oxidative unsymmetrical cleavage of the azo linkage.

On the contrary, azobenzene, 4-nitroazobenzene, and 4-phenylazo-benzoic acid did not react. Instead, a noticeable catalyst autodestruction was observed. Preliminary work on Sudan II showed that this dye was bleached upon oxidative unsymmetrical azo linkage cleavage leading to a phenylazene and 1,2-naphthoquinone and their further degradation products (Scheme 11).

**Scheme 12:**
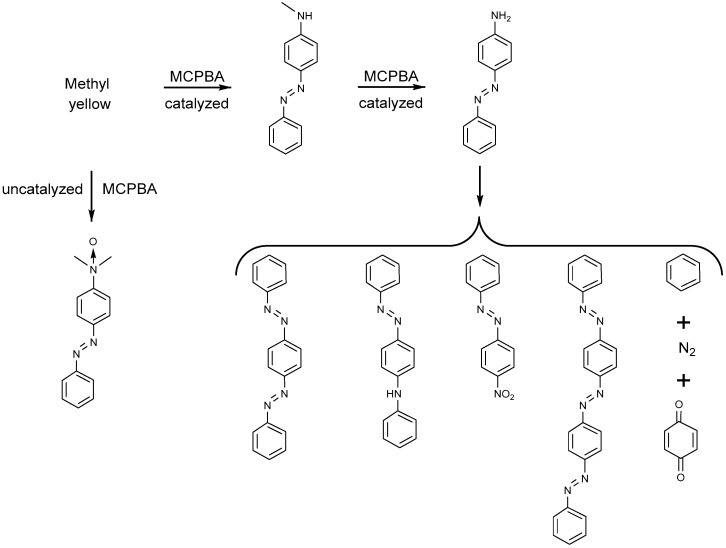
Fe(TDCPP)Cl/MCPBA system in the case of azo dyes with *N*,*N*-dimethylamino substituent gives rise to gradual demethylation, followed by oxidation, leading to a complex mixture of products.

In the case of amino-containing azo dyes, to a certain extent such a pathway is still operating (with the obvious difference that a quinoneimine rather than a quinone is the primary product of the cleavage reaction). However, the main pathway passes through a direct attack of the oxidizing system to the amine nitrogen of the dyes. In the case of Methyl Yellow, which contains a *N*,*N*-dimethylamino substituent, this was gradually demethylated and oxidized, giving rise to a complex mixture of different products, as shown in Scheme 12. By this pathway the azo linkage remains untouched, and further azo linkages are formed through well-known condensation reactions [196,197,198,199], giving rise to polyazo, quite recalcitrant dyes. Interestingly, iron(III) porphyrin promoted oxidation of amino azo dyes can lead to nitro azo dyes, representing dead end products of the reaction and posing additional toxicological and environmental concerns. As a point of fact, peroxyacid action towards dialkylamino azo dyes (such as Methyl Yellow and Methyl Orange) in the absence of iron(III) porphyrin catalysts affords the corresponding *N*-oxides. These lack the extensive electron conjugation of the parent azo dyes, and so appear deceptively pale-colored in comparison to their azo counterparts. This is an outstanding example of how the simple decolorization criterion could be unsatisfactory to assess the bleaching of a given azo dye.

Another cationic porphyrin, the iron(III) complex of 5,10,15,20-tetrakis(*N*-methylpyridinium-4-yl)porphyrin, immobilized by ionic exchange on montmorillonite K10, was applied as a catalyst in Disperse Orange 3 (4-(4′-nitrophenylazo)aniline) oxidation by hydrogen peroxide [141,200]. In that study, pH 3 was optimal for dye degradation, which mainly gives 4-nitroaniline as the oxidation product. In particular, 4-nitroaniline became almost the sole product when *tert*-butylhydroperoxide was substituted for hydrogen peroxide as the oxidant. However, when iodosylbenzene was used, no asymmetrical cleavage of the azo linkage was observed, and the main product was 4-(4′-nitrophenylazo)nitrobenzene. Somewhat surprisingly, iodosylbenzene, which is an oxygen donor capable of directly generating the *Cpd I* analog, proved to be incapable of cleaving the azo linkage. On the whole, the catalytic system–which strictly resembles the action of hydrogen peroxide in the presence of high-redox-potential peroxidases [201]—is not able to transform the toxic dye Disperse Orange 3 in less dangerous compounds. In fact, 4-nitroaniline and in particular 4-(4′-nitrophenylazo)nitrobenzene are very toxic and recalcitrant aromatic compounds.

In conclusion, amino azo dyes, although attacked by oxidants in the presence of iron(III) porphyrin catalysts, give rise to major amounts of oxidation and/or condensation products, where the azo moiety is kept intact, and showing both increased recalcitrancy and toxicity in comparison with their parent dyes.

Mn(III) porphyrins have been the focus for a number of studies where some have proven to be very effective bleaching catalysts as discussed in Section 2. Pioneering experiments [202] dealing with the Mn(III) complex of 5,10,15,20-tetraphenylporphyrin, complexed with imidazole, as a catalyst in industrial wastewaters containing both azo dyes and detergents, as well as perborate bleach, showed that the system was poorly effective. Bleaching effectiveness was still decreased by detergents, masking dye molecules within micelles, therefore preventing the approach of the high-valent metalloporphyrin intermediates, responsible for dye oxidation. Sulfonation of the porphyrin has only a marginal effect towards catalyst effectiveness. Moreover, perborate showed an irreversible, destructive action against the catalyst itself, when dye substrates were absent. The authors concluded that at least the Mn(III) porphyrin they studied is not promising as an activator for azo dye bleaching in industrial wastewaters also containing—as is usual—detergents.

**Figure 6:**
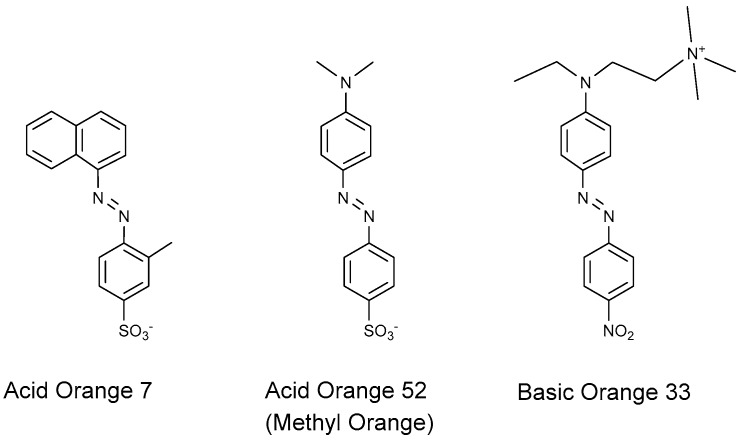
Three azo dyes (Acid Orange 7, Acid Orange 52, and Basic Orange 33) were used as the substrates in the first study about bleaching by Mn(III) porphyrins.

The first study on azo dye bleaching by H_2_O_2_ in the presence Mn(III) porphyrins was conducted by using the catalysts covalently bound to a PEG chain to ensure solubility in water under mild experimental conditions (25 °C, pH 7 or 8) [203]. Three azo dyes (Acid Orange 7, Acid Orange 52, and Basic Orange 33, Figure 6) were tested as substrates for decolorization experiments. At first, catalysts stability was tested in the presence of hydrogen peroxide and in the absence of dye substrates: the Mn(III) porphyrins, where the eight β positions had been substituted with bromine, proved to be quite resistant against destruction by the oxidant, whereas unsubstituted porphyrins were largely destroyed within a few hours. However, such a resistance against bleaching by hydrogen peroxide did not correlate with catalytic efficiency, as the Mn(III) β-octabromoporphyrins were poorer catalysts than their unsubstituted counterparts. This is in agreement with previous studies, where the adverse effect of bulky substituents (such as bromine) at the β positions has been underlined. On the whole, the MnTDCP series was more effective as decolorization catalyst than MnTPFPP. The catalytic performances of the studied Mn(III) porphyrins was significantly enhanced in the presence of imidazole: this favored the heterolytic cleavage of the *Cpd 0* and thus the formation of the very effective *Cpd I* analog over the milder oxidant *Cpd II* analog. The bleaching kinetics of the decolorization was also studied, and a noticeable resemblance of the catalysts behavior with that of their enzymatic counterpart was pointed out, therefore allowing the use of the same format, comprising *K_M_* and *k_cat_*.

More recently, the action of similar PEG-bearing Mn(III) porphyrins was studied on the water-insoluble dye Solvent Yellow 14 in non-aqueous solvents such as benzene, chloroform, dichloromethane, pyridine, dimethylsulfoxide, tetrahydrofuran, and methanol [204]. Interestingly, in the presence of imidazole the bleaching efficiency decreased, opposite to that observed in an aqueous environment. Such a surprising behavior perhaps depends on a *bis*-coordination of the Mn(III) ion within the porphyrin ring, favored by water absence.

In another study, two manganese(III) porphyrins, namely the Mn(III) complexes of 5,10,15,20-tetrakis(4-carboxyphenyl)porphyrin and 5,10,15,20-tetrakis(*N*-methylpyridinium-4-yl)porphyrin, were used to catalyze the bleaching of the azo dyes Acid Orange 7 and Basic Orange 33 by hydrogen peroxide, under very mild conditions (25 °C and pH 8) [205]. Imidazole addition caused substantial increase in the catalytic efficiency, most probably because it favors the formation of high-valent manganese intermediates such as PorphMn(IV)=O or PorphMn(V)=O, which are the causal agents of dye bleaching. No significant differences in the bleaching rates were observed with both the Mn porphyrins when the anionic and the cationic dyes were exchanged. This observation suggests that under the experimental conditions adopted, no noteworthy importance should be assigned to electrostatic attractive or repulsive effect with respect to bleaching mechanism.

**Scheme 13:**
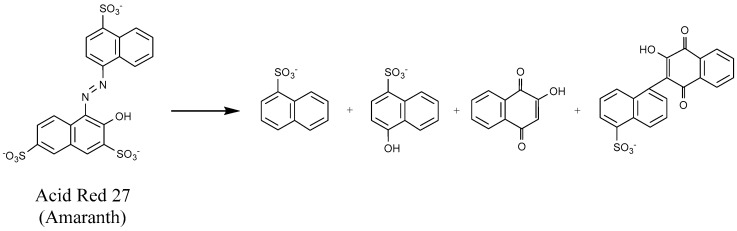
Acid Red 27 (amaranth) is cleaved by Mn porphyrins in various products, such as the salts of naphthalene-1-sulfonic acid, 1-naphthol-4-sulfonic acid, and 2-hydroxy-1,4-naphthoquinone (lawsone), concomitantly to the formation of a dimer.

Acid Red 27 (amaranth, Scheme 13), once used as a food dye but withdrawn for its noticeable toxicity, has been recently studied [140] as the substrate for oxidation by H_2_O_2_ in aqueous solution, in the presence of the Mn(III) complex of 5,10,15,20-tetrakis(1-methylpyridinium-4-yl) porphyrin MnT4MPyP. First, the mechanism of hydrogen peroxide with this cationic Mn porphyrin was studied. The *Cpd 0* analog rapidly changed into a *trans*-dioxo-Mn(V) derivative (a *Cpd I* analog), which in turn slowly fades, affording a *trans*-dioxo-Mn(IV) derivative (a *Cpd II* analog). When Amaranth was present, the *Cpd I* analog was rapidly reduced to its *Cpd II* analog counterpart, whereas the dye was bleached. The *Cpd II* analog is strong enough as an oxidizing agent to oxidize another dye molecule. Various cleavage products were found, such as naphthalene-1-sulfonic acid, 1-naphthol-4-sulfonic acid, and 2-hydroxy-1,4-naphthoquinone (lawsone). The optimum pH for the bleaching reaction was found at 10.4. The formation of an inactive complex between the deprotonated dye and the catalyst under stronger alkalinity was suggested.

**Figure 7:**
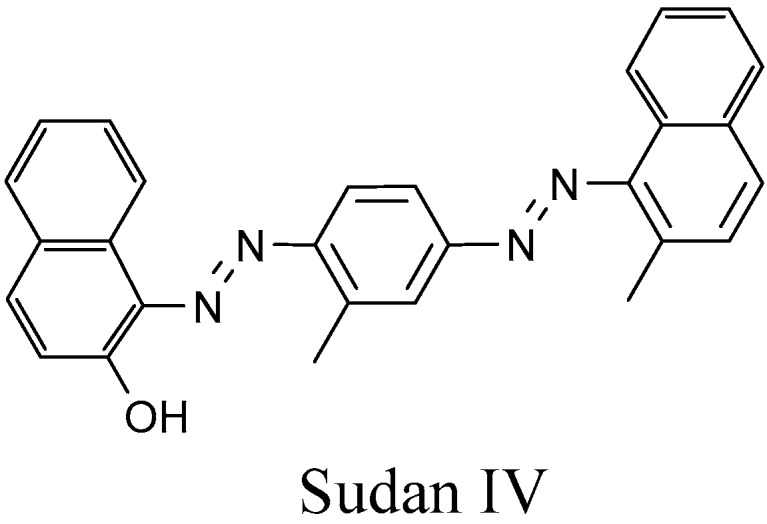
Methyl Orange (please, refer to Figure 6) and Sudan IV have been the subject of a comparative bleaching study by dichloro- or dibromophenyl *meso*-substituted Mn porphyrins.

In another study some Mn porphyrins were tested as catalysts for azo dye oxidation (Sudan IV and Methyl Orange, Figure 7) [206]. Sudan IV bleaching experiments were carried out in dichloromethane solution, whereas Methyl Orange was tested in aqueous solution. The chosen porphyrins were the Mn(III) complexes of TDCPP, 5,10,15,20-tetrakis(2,4,6-tribromophenyl)porphyrin, 5,10,15,20-tetrakis(2,4,6-tribromo-3-methoxyphenyl)porphyrin, 5,10,15,20-tetrakis(2,4,6-tribromo-3-*N*,*N*-diethylsulfonamidophenyl)porphyrin, and 5,10,15,20-tetrakis(2,4,6-tribromo-3-*p*-toluene-sulfonyl-oxyphenyl)porphyrin.

A deep comparative study was planned, where some parameters were varied to find the best conditions for Methyl Orange bleaching (Sudan IV was judged as less interesting owing to its insolubility in water). Halogen substitution at the *meso*-phenyls proved to be advantageous, as shown by the observation that simple Mn complex of *meso*-tetraphenylporphyrin was the worst catalyst when compared to its halogenated derivatives. The best catalyst was the dichlorosubstituted one, bearing an additional diethylsulfonamide moiety at the 3′ position. The addition of *tert*-butylpyridine as a co-catalyst was found to be sharply favorable to catalyst efficiency.

On the other hand, azo dyes could successfully resist against bleaching, mediated by Mn porphyrins, as shown by an interesting study where aromatic amines were oxidized with high yields to the corresponding azo dyes (through oxidative coupling) by periodate, in the presence of the Mn(III) complex of *meso*-tetraphenylporphyrin and some imidazole and pyridine axial ligands for the metal center [207]. However, under proper experimental conditions, some Mn porphyrins efficiently oxidize and bleach azo dyes, as reported above. In particular, in a biphasic, micellar system [208] the water insoluble azo dye Sudan IV was bleached by hydrogen peroxide in the presence of two manganese porphyrin complexes, namely 5,10,15,20-tetrakis(dichlorophenyl)porphyrin and 5,10,15,20-tetrakis(2,4,6-tribromo-3-methoxyphenyl)porphyrin, in the presence of the activators *tert*-butylpyridine and benzoic acid. More recently, the same Mn porphyrins and two other catalysts containing sulfonate moieties at the four peripheral phenyl rings in addition to the two chlorine and the three bromine substituents, were tested to assess their catalytic activity in Methyl Orange bleaching by H_2_O_2_ [206]. Additionally, these four catalysts were compared with the manganese complex of 5,10,15,20-tetraphenylporphyrin, which was by far the worst, as expected. The experiments were carried out in a dichloromethane/water biphasic system, and showed the high catalytic efficiency of the Mn porphyrins tested, although they stayed in the organic phase, whereas Methyl Orange was in the aqueous phase. Unfortunately, the authors gave no data about the degradation products of Methyl Orange under the experimental conditions tested.

In conclusion, azo dyes bearing aromatic amine functions could be oxidized by hydrogen peroxide or other suitable oxidants in the presence of Fe or Mn porphyrins. However, noticeable amounts of complex products, still containing the azo moiety and carrying the nitro function arise, being more toxic and recalcitrant than the starting dyes. This drawback is not observed with phenolic azo dyes that undergo oxidative cleavage at the azo function, therefore giving elemental nitrogen together with quinones and other hydroxylated aromatic fragments.

#### 4.1.2. Anthraquinone Dyes

Although anthraquinone dyes are widespread for several applications, in-depth studies about their oxidative degradation, possibly by enzymatic or chemical catalysis are rare. In particular a detailed report has been published [209] dealing with alizarin (1,2-dihydroxyanthraquinone) degradation by H_2_O_2_ under strongly alkaline conditions. The transient formation of an epoxy intermediate along the degradation process was suggested. On the other hand, anthraquinone dye degradation has been the target of several studies, usually focused on the use of titania as the photocatalytic agent [210,211,212]. Also electrochemical methods have been described [213,214]. A detailed study was conducted on Alizarin Red S (ARS) degradation by H_2_O_2_ in the presence of the manganese complex of 5,10,15,20-tetrakis(4-sulfonatophenyl)porphyrin (TSPP), coordinatively immobilized on a silica matrix, functionalized with covalently bound *N*-propylimidazole moieties [144]. The catalyst was optimally effective at pH 7. Decolorization was more than 50% after 2 h, whereas the dye was totally bleached within 3 h.

**Scheme 14:**
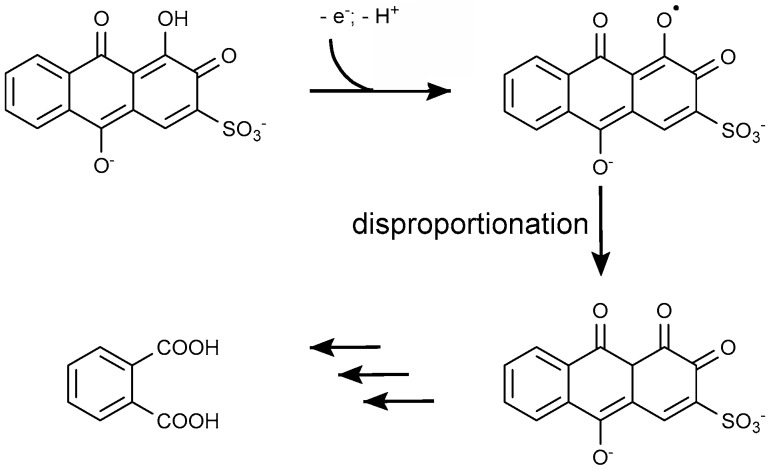
Alizarin Red S is bleached by a Mn porphyrin complex through a HAT mechanism leading to incomplete mineralization.

In principle, two alternative mechanisms could be suggested to describe the oxidative bleaching of the dye: (a) oxygen transfer (direct or through a rebound mechanism) from the *Cpd I* analog to ARS, targeted to the double bond shared between the sulfocatechol and the quinone rings; and (b) an HAT (or ET, depending on pH) mechanism, passing through a *Cpd II* analog. A third alternative, involving hydroxyl radical intermediacy, was ruled out thanks to two findings: ARS successfully resisted against heating in the presence of the well-known ∙OH generator azo-*bis*-propionamidine dihydrochloride (APH), and the degradation was unaffected by the addition of mannitol, a well-known ∙OH scavenger. The oxygen transfer would lead to a transient, very reactive epoxide, so paralleling that described for alizarin bleaching by hydrogen peroxide under strongly alkaline conditions [209]. However, the close similarity of the UV/Vis bleaching pattern between the described bleaching and the laccase-catalyzed one (*vide infra* Section 4.3) strongly speaks in favor of a HAT mechanism, which has been detailed (Scheme 14). The degradation of the dye is not a complete mineralization, as the unsubstituted ring of the original molecule was found after bleaching completion as *o*-phthalic acid.

**Figure 8:**
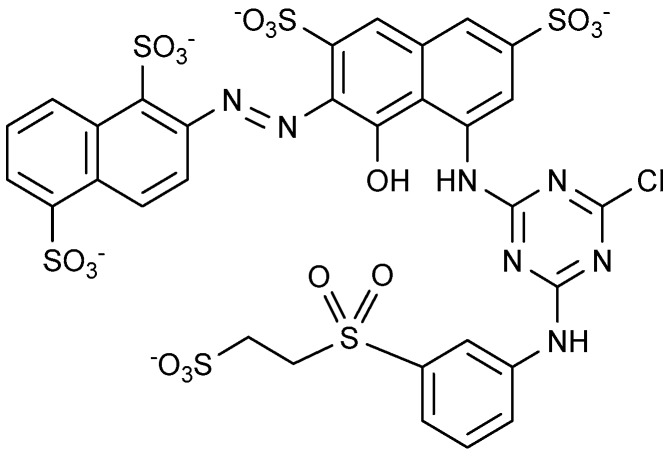
Reactive Red 195 structure.

Hematin, a naturally occurring Fe(III) porphyrin known to mimic peroxidases, has been proposed as a catalyst for ARS and Orange II bleaching by H_2_O_2_ [215]. Hematin was used as an immobilized preparation on to glutaraldehyde-activated chitosan beads, also in the presence of aminopropyltriethoxysilane. The authors hypothesized the formation of covalent linkages between glutaraldehyde and hematin, eventually with aminopropyltriethoxysilane intermediacy. In another study [216], hemin (ferriheme) was used in combination with hydrogen peroxide to bleach some synthetic azo dyes such as Reactive Red 195 (Figure 8). Activity and stability of the catalyst were substantially improved upon adsorption on to carbon fibers.

**Scheme 15:**
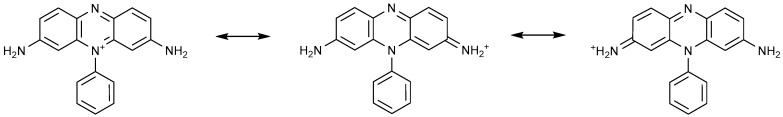
Phenosafranine structure.

Although poorly used, phenosafranine (Scheme 15) is a toxic, polluting, and recalcitrant dye, whose inexpensive and “green” degradation could be of interest. Accordingly, in a recent study it was tested as a substrate for a bleaching oxidation by H_2_O_2_, under catalysis by silica-supported MnTSPP [143].

#### 4.1.3. Cationic Dyes

Phenosafranine belongs to cationic dyes, showing an *N*-phenylphenazinium delocalized cation also bearing two peripheral amino groups. Therefore, the compound is an electron-deficient heteroaromatic, quite resistant to attack by electrophilic agents. Consequently, the only described chemical bleaching procedure for phenosafranine uses 0.5 M potassium peroxodisulfate under strongly acidic conditions and leads to dye polymerization and precipitation rather than bleaching. The reaction goes through a two consecutive one electron oxidation, producing a radical dication and a nitrenium dication, respectively [217]. The latter is an extremely powerful electrophile, triggering a polymerization process at the expenses of the still unreacted phenosafranine molecules. Obviously, such a treatment is not applicable for wastewater remediation, and a milder procedure is of potential usefulness. The study showed that in the presence of the above catalyst, H_2_O_2_ at a concentration as low as 8.8 mM efficiently and completely bleached the dye (starting concentration 0.3 mM) within 3 h. As a point of fact, 8.8 mM peroxodisulfate was quite inert towards 0.2 mM phenosafranine, and the same was observed when working with 8.8 mM H_2_O_2_ in the absence of MnTSPP catalyst. With concern to the reaction mechanism, the H_2_O_2_/catalyst system most probably acts differently from peroxodisulfate, as revealed by the different UV/Vis spectroscopic patterns observed for the two oxidants. Perhaps an OT mechanism operates, possibly together with an ET/HAT one, provided that the Mn-based *Cpd I* analog is stable enough to significantly oxygenate susceptible substrates prior to fade by ET/HAT to the less reactive *Cpd II* analog.

**Figure 9:**
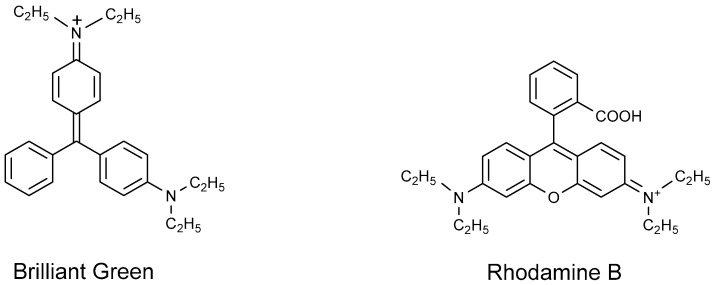
Brilliant Green (a triphenylmethane) and Rhodamine B (a tricyclic immonium/oxonium cation) structures.

Another cationic triphenylmethane dye, Brilliant Green (as the hydrogen sulfate, Figure 9) has been extensively studied [218] as a substrate for bleaching by hydrogen peroxide in water, under catalysis by two similar metalloporphyrins, Mn(III)T4MPyP, and the Mn(II) complex of β-octabromo-5,10,15,20-tetrakis-(*N*-methylpyridinium-3-yl)porphyrin (Br_8_T3MPyP).

The latter is an uncommon example of a low-spin Mn(II) complex, favored by the electron-withdrawing features of both bromine and pyridinium substituents on to the porphyrin ring. The catalysts were immobilized by ion exchange onto two different supports: halloysite nanotubes and silica-coated magnetite nanoparticles. As a general result, Mn(III)T4MPyP performed better than the other metalloporphyrin, owing to the presence of eight bulky bromine substituents on the latter, which prevent the optimal approach of the substrate to the metal center. Moreover, magnetite-based solid support seems to be more promising since it allows catalyst reuse (whereas halloysite caused a rapid drop in catalyst activity after the first use) and recovery by means of an external magnetic field.

#### 4.1.4. Applications of Metalloporphyrins as Generic Decolorizing Catalysts

A number of published studies are devoted to the assessment of the usefulness of redox-active metalloporphyrins as “generic” decolorizing catalysts rather than to the study of bleaching mechanisms of selected dyes. Among these, a comparative study involving two metalloporphyrin catalysts, namely the manganese and iron complexes of 5,10,15,20-tetrakis(4-carboxyphenyl)porphyrin (TCPP), acting as promoters of oxidative bleaching of four dyes by H_2_O_2_ has been reported [185]. The tested dyes were Methyl Orange, Crystal Violet, Methylene Blue, and Congo Red: azo, triphenylmethane, and thiazine dye classes were therefore represented. The catalysts were covalently immobilized on multi-walled carbon nanotubes (MWCNTs). Both catalysts showed their best performances at slightly acidic pH values (around 5), being the Fe-based catalyst more active towards Methyl Orange than its Mn-based counterpart. By contrast, the Mn-porphyrin is more active towards the other dyes. The presence of imidazole cocatalyst was compulsory to observe significant catalytic activity in the case of the Mn-porphyrin, whereas it completely inhibited the Fe-porphyrin activity.

In another comparative study, six textile dyes, namely Alizarin Red S, Phenosafranine, Xylenol Orange, Methylene Blue, Methyl Green, and Methyl Orange, were tested as substrates for oxidative bleaching in the presence of two catalysts [162]: MnTSPP immobilized on imidazole-functionalized crosslinked polyvinyl alcohol, and FeTPFPP immobilized on pyridine-functionalized silica gel. Such organic functions behaved as ligating agents for Mn(III) and Fe(III) within the porphyrin macrocycles. Bleaching was studied at pH 7, in aqueous environment, without any added organic cosolvent, and the bleaching agent was H_2_O_2_. The results showed that the Mn-based catalyst was sharply more effective, whereas the Fe-based catalyst needed a redox mediator such as Mn^2+^ ion complexed with malonate to prevent disproportionation of the active species, Mn(III). The same preparation FeTPFPP immobilized on crosslinked pyridine-functionalized PVA was used to oxidatively bleach a number of organic compounds with H_2_O_2_ [31]; in particular, the decolorization of the thiazine dye Azure B was studied under different experimental conditions. This dye was chosen because it is an ideal substrate for LiP, but not for MnP [219]. However, a positive effect of Mn(II)/malonate addition to the reaction mixtures has been reported for that study.

**Figure 10:**
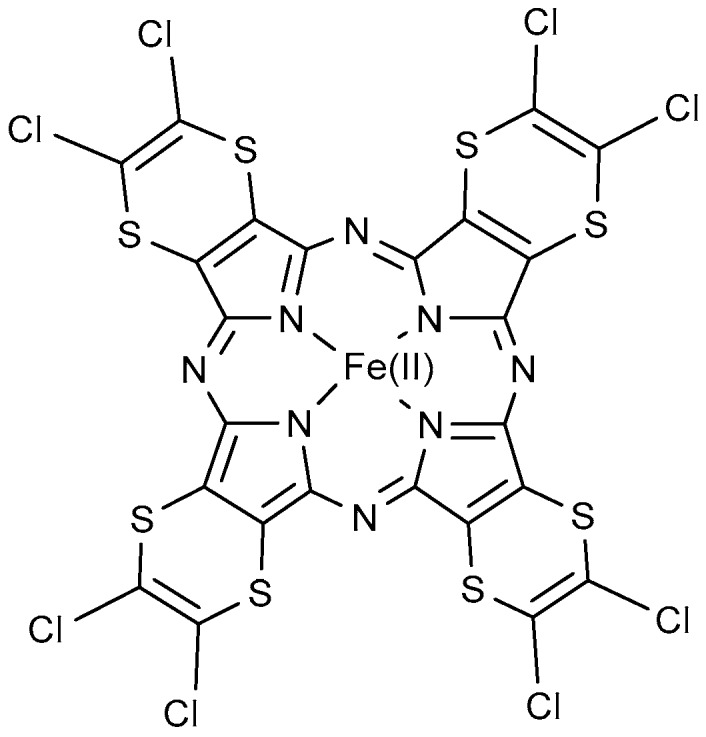
Iron(II) tetrakis(5,6-dichloro-1,4-dithiin)porphyrazine is a catalyst strictly resembling the structure of “normal” metalloporphyrins.

Another synthetic catalyst, iron(II) tetrakis(5,6-dichloro-1,4-dithiin)porphyrazine (Figure 10), strictly resembling in its structure the “normal” metalloporphyrins, has been studied [220] to assess its ability in catalyzing the degradation of the cationic dye Rhodamine B (a tricyclic oxonium cation) by hydrogen peroxide (Figure 9). Formic, acetic, benzoic, and *o*-phthalic acids were detected among the degradation products of the dye. The reaction mechanism was different at pH 2 (formation of a *Cpd I* analog) and pH 7 (homolytic fission of the O–O bond of the *Cpd 0* analog, with concomitant ∙OH production, as assessed by means of EPR analysis).

Although not directly related to industrial dye bleaching an outstanding study must be cited here. It is the case of the Mn(III) complex of 5,10,15,20-tetrakis(1,3-dimethylimidazolium-2-yl)porphyrin (TDMImP) which proved to be capable of evolving chlorine dioxide from chlorite in aqueous solution at slightly acidic pH values and at about room temperature [221]. Somewhat surprisingly, the apparently similar Mn(III)TMPyP [both (*N*-methylpyridinium-2-yl) and (*N*-methylpyridinium-4-yl) isomeric forms) were quite inactive, perhaps owing to their too high redox potentials (Mn(V)/Mn(III) couples) [222] to be oxidized by ClO_2_^−^. The authors have proposed a reaction cycle that satisfactorily explains the ClO_2_ production. The reaction was studied both in solution and with the catalyst adsorbed onto montmorillonite; in both cases a transient O=Mn(V)=O *trans*-dioxo species was found. If not removed, the formed ClO_2_ slowly reduced the Mn(V)porphyrin to its Mn(IV) counterpart, while it is changed into chlorate ClO_3_^−^. The obtained ClO_2_ could be easily stripped from the solution by sparging it with air, and then bubbled within the to-be-bleached solution. Chlorine dioxide is poorly studied as a dye-bleaching agent [223] due to its very unstable nature: it can explode and has to be generated close to the plant where has to be used. However, taking into account its very strong oxidizing properties and its noticeable tendency to oxidize organics with no concomitant chlorination, its potential as a very effective bleaching agent should be considered.

### 4.2. Effect of Redox Mediators

In the case of redox-active enzymes, and in particular for fungal laccases, the use of redox mediators (RMs) to widen the application field of these enzymes towards compounds that are not per se laccase substrates, is well-known [224] and has been recently reviewed [169]. Therefore, it seems obvious to extend the use of such compounds to other oxidative enzymes and to their emulators, redox-active metalloporphyrins. Somewhat unexpectedly, only a few articles have been published about the use of RMs together with such catalysts, and the reports underline the substantial lack of activity of these putative co-catalysts. What are the reasons for these disappointing results? First of all, one should note that the redox potentials of the commonly used metalloporphyrins are, as a rule, higher than those observed for fungal laccases (that are the most powerful oxidizing agents among the laccases in general) [225]. Therefore, added RMs cannot help redox-active metalloporphyrins to expand their substrate inventory on the sole basis of electrochemical considerations: on the contrary, an adverse effect could be anticipated, as a superfluous additional step is interposed between the high-valent oxometal center of the metalloporphyrin and the to-be-oxidized substrate. On the other hand, some RMs could be helpful when the shape and/or the bulky nature of the substrates hinders or also totally preclude the proper approach of the substrate to the catalytic center. This could be most probably the case of the industrial dye Methyl Green when bleached by H_2_O_2_ under catalysis by MnTSPP immobilized on imidazole functionalized silica gel [162]. Regardless to the particular RMs used (TEMPO, *N*-hydroxysuccinimide, *N*-hydroxy-phthalimide, *N*-hydroxybenzotriazole) the activity of the catalyst was about tripled, whereas other dyes under the same conditions gave contrasting results: for example, in the case of Methyl Orange the addition of TEMPO produced a dramatic drop in catalyst activity, and in the case of phenosafranine the catalyst activity was invariably worsened by the four RMs tested [143]. In this last case, the electrochemical explanation as above is the most likely. In the case of Alizarin Red S under the same experimental conditions [144] the same RMs were ineffective (TEMPO and *N*-hydroxyphthalimide) or produced a moderate increase in bleaching effectiveness (*N*-hydroxysuccinimide and *N*-hydroxybenzotriazole). The reasons of such behaviors are unknown.

Starting from the well-known occurrence of hemoenzymes specifically featured by Nature to oxidize Mn(II) to Mn(III) chelates (fungal manganese peroxidases, MnPs), Mn(II) salts in the presence of a sodium malonate buffer (as a chelating and stabilizing agent for the putative Mn(III) species arising from oxidation) have been sometimes tested to assess the putative MnP emulating ability of some redox-active metalloporphyrin preparations. When FeTPFPP was immobilized on pyridine-functionalized cross-linked PVA [30], Mn(II) addition slightly enhanced catalyst performances above pH 6, whereas the opposite was observed at lower pH values. The reasons for this behavior are unknown. As a point of fact, when veratryl alcohol (a widely accepted simple model compounds to evaluate ligninolytic activity) was used as the substrate, its influence was invariably favorable. However, it should be noted that the catalytic activity was evaluated in terms of veratraldehyde production, whereas it was found that the studied FeTPFPP produced mainly 2-hydroxymethyl-5-methoxy-1,4-benzoquinone as the oxidation product of veratryl alcohol [31]. Therefore, the observed increased production of veratraldehyde is not necessarily to be ascribed to a true activity enhancement. In other words, the presence of the manganese salt could drive the reaction to the aldehyde at the expenses of substituted benzoquinone formation. In conclusion, also the RMs couple Mn(II)/Mn(III) has found no significant application in the field of technological use of biomimetic or bioinspired redox-active metalloporphyrins.

### 4.3. Comparison with Enzymatic Decolorization

Several enzymes have been also used in the bleaching of textile dyes [226,227,228,229,230], including in particular laccases and peroxidases. Their comparison is crucial to encompass the catalytic potential of immobilized metalloporphyrins since usually enzymatic catalysts stand out about efficiency [146,231,232,233,234] and specificity [29,231]. In particular, the use of high-potential fungal laccases has been recently discussed [169,235], being this enzyme able to oxidize a wide range of natural and synthetic phenolics and aromatic amines [236,237,238,239,240].

Several studies have shown the ability of microbial laccases to specifically bleach industrial azo dyes [241,242]. Laccases cannot produce any direct oxygenation of the substrates, but only oxidize them by ET/HAT mechanisms, depending on pH, substrate structure, and other operational conditions. In this sense, they somewhat resemble “classical” peroxidases. Therefore, in the presence of some electron-withdrawing substituents in the azo dye structure, a noticeable recalcitrancy towards oxidative degradation could be easily anticipated, and such a forecast is met by the experimental work. Laccases could bleach many dyes, not belonging to the azo dye class [243,244,245,246], possibly in the presence of suitable redox mediators [247,248].

Owing to its relatively low redox potential, horseradish peroxidase is not the tool of choice to build a versatile bleaching system for industrial dyes. However, in some selected cases it represents an inexpensive and effective alternative to more complex and costly procedures [227,249,250]. Only seldom can horseradish peroxidase act as an oxygen donor towards some substrates [251]. As a point of fact, horseradish peroxidase was inactive towards ARS, whereas a moderate activity was found when using *Pleurotus pulmonarius* (formerly referred to as *P. sajor-caju*) laccase. However, bleaching with laccase was incomplete, perhaps because of the formation of a 1,2,9,10-anthradiquinone-3-sulfonic acid as an intermediate, which slowly fades and probably has an adverse effect on the enzyme. Anyway, the spectrophotometric patterns of the two processes (H_2_O_2_ and MnTSPP versus laccase) are qualitatively similar, which suggests a similar degradation pathway passing through the diquinone intermediate. Only in the case of the artificial catalyst the reaction goes forward leading to *o*-phthalic acid as the main degradation product. Not surprisingly, H_2_O_2_ addition to laccase enhances the bleaching process, although it becomes not as efficient as observed with the system hydrogen peroxide plus metalloporphyrin.

The things go differently in the case of high-potential peroxidases such as LiP, MnP, and haloperoxidases: the two azo dyes Orange G and Sunset yellow were efficiently bleached by hydrogen peroxide in the presence of very low concentrations of chloroperoxidase [189]. Two alternative cleavage mechanisms were identified by studying the product pattern with LC-MS (ESI): a symmetrical one (the double bond of the azo chromophore is cleaved) and two variants of unsymmetrical breaking, each involving one linkage between the azo chromophore and one of the two aromatic moieties. Similar conclusions were reached (coexistence of symmetrical and unsymmetrical cleavage mechanisms) in the case of MnP acting on Orange II [252]. However, also high-potential peroxidases sometimes fail in bleaching recalcitrant dyes. This is the case of phenosafranine: the dye was at first partially bleached by H_2_O_2_ in the presence of LiP, but the enzyme was rapidly and irreversibly inactivated along the bleaching process [143]. Perhaps a very reactive intermediate (the same hypothesized for the metalloporphyrin-catalyzed bleaching?) attacks the enzyme leading to permanent modification and therefore loss of catalytic activity.

## 5. Conclusions

Redox-active metalloporphyrins are very promising bleaching catalysts for synthetic textile dyes when the ecofriendly and not too costly hydrogen peroxide is used as the oxidizing agent. However, they also show some limitations and drawbacks to be kept in mind when envisaging a specific bleaching process. First of all, metalloporphyrins are quite expensive compounds, which have to be recovered and recycled to build any economically sustainable process. This goal can be achieved by immobilization on proper supports, recoverable by means of decantation, filtration, or by means of applied magnetic fields. Metalloporphyrin toxicology is largely unknown, and therefore efficient recovery of the catalysts is even more compulsory. Adverse effects can be easily forecast, owing to their resemblance to physiological metalloporphyrins such as heme; the topic is quite complex and is far from being deeply explored [253,254,255]. Another limitation has been discussed above, and is related to amino azo dyes, which when attacked by metalloporphyrins produce compounds that are often more toxic and recalcitrant than the original dyes. On the other hand, the lack of specificity and the high redox potentials of the metalloporphyrins allow the efficient bleaching of a wide range of different dyes, almost regardless to the specific chemical classes. This is a very useful feature when working on dyes, whose bleaching by means of oxidative enzymes is not possible, as well as depicted by the example of phenosafranine. In conclusion, some guidelines can be proposed to enhance the usefulness of redox-active metalloporphyrins to remediate textile wastewaters: (i) exploring the (inexpensive) synthesis of more and more active and stable catalysts; (ii) envisaging new supports and new procedures to obtain robust and effective immobilized catalysts; (iii) optimizing the operative conditions to balance higher bleaching rates, and lower oxidant consumption with minimal catalyst degradation.

## Figures and Tables

**Table 1:** Typical Physical chemical composition of textile wastewaters [1,12,20,21,22,23,24].

Parameter	Range
pH	5–13
Temperature (°C)	20–60
Conductivity (mS/cm)	0.1–120
Biological Oxygen Demand (ppm)	14–6000
Chemical Oxygen Demand (ppm)	150–90,000
Total Suspended Solids (ppm)	100–25,000
Total Dissolved Solids (ppm)	1800–30,000
Total Kjeldahl Nitrogen (ppm)	70–160
Turbidity (NTU)	0–200
Color (Pt-Co)	50–2500
Absorbance	0.9–200

**Table 2:**
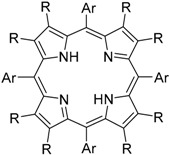
*meso*-Tetrasubstituted porphyrins with and without substituents in the β-positions.

Acronyms	Designations	Ar	R
TPP	5,10,15,20-tetraphenylporphyrin	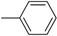	8 H
TDCPP	5,10,15,20-tetrakis(2,6-dichlorophenyl)porphyrin	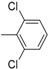	8 H
TDCPCl_8_P	β-octachloro-5,10,15,20-tetrakis(2,6-dichlorophenyl)porphyrin	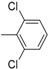	8 Br
TPFPP	5,10,15,20-tetrakis(pentafluoro-phenyl)porphyrin	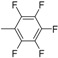	8 H
TPFPF_8_P	β-octafluoro-5,10,15,20-tetrakis(pentafluorophenyl)porphyrin	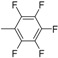	8 Br
TCPP	5,10,15,20-tetrakis(4-carboxy-phenyl)porphyrin	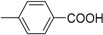	8 H
TMCPP	5,10,15,20-tetrakis(4-methoxy-carbonylphenyl)porphyrin	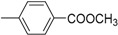	8 H
THPP	5,10,15,20-tetrakis(4-hydroxy-phenyl)porphyrin	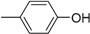	8H
TDCSPP	5,10,15,20-tetrakis(2,6-dichloro-3-sulfonatophenyl)porphyrin	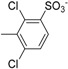	8 H
T4MPyP	5,10,15,20-tetrakis(*N*-methyl-pyridinium-4-yl)porphyrin	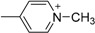	8 H
TBr_3_MPP	5,10,15,20-tetrakis(2,4,6-tribromo-3-methoxyphenyl)porphyrin	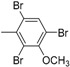	8 H
TSPP	5,10,15,20-tetrakis(4-sulfonato-phenyl)porphyrin	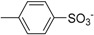	8 H
Br_8_T3MPyP	β-octabromo-5,10,15,20-tetrakis-(*N*-methylpyridinium-3-yl)porphyrin	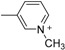	8 Br
TDMImP	5,10,15,20-tetrakis(1,3-dimethyl-imidazolium-2-yl)porphyrin	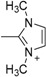	8 H

**Table 3:** Main features of the various immobilization approaches for metalloporphyrins [29].

Type of Interaction	Method of Immobilization	Advantages	Disadvantages
Physical inclusion	Encapsulation/entrapment	Minimization of chemical modification of the catalyst	Leakage
			No emulation of peroxidases active site
			Involvement of toxic and costly reagents
Weak chemical interaction	Adsorption	Minimization of chemical modification of the catalyst	Usually the interaction catalyst/support is weak
		Easy method	Leakage
			No emulation of peroxidases active site
	Ion exchange	Minimization of chemical modification of the catalyst	Usually the interaction catalyst/support is weak
			Leakage
			No emulation of peroxidases active site
Strong chemical interaction	Covalent binding	The interaction catalyst/support is very strong	Chemical modification of the catalyst is possible
		Minimal catalyst leakage	No emulation of peroxidases active site
	Axial coordination	Real emulation of peroxidases active site	Axial *bis*-ligation of the catalysts
		Increased stability and activity	
